# Genetic characterization of *Strongyloides fuelleborni* infecting free-roaming African vervets (*Chlorocebus aethiops sabaeus*) on the Caribbean island of St. Kitts

**DOI:** 10.1016/j.ijppaw.2023.02.003

**Published:** 2023-02-16

**Authors:** Travis Richins, Sarah G.H. Sapp, Jennifer K. Ketzis, Arve Lee Willingham, Samson Mukaratirwa, Yvonne Qvarnstrom, Joel L.N. Barratt

**Affiliations:** a Centers for Disease Control and Prevention, Division of Parasitic Diseases and Malaria, Parasitic Diseases Branch, USA; b Oak Ridge Associated Universities, Oak Ridge, TN, USA; c Biomedical Sciences, One Health Center for Zoonoses & Tropical Veterinary Medicine, Ross University School of Veterinary Medicine, Saint Kitts and Nevis; d Department of Veterinary Medicine, College of Agriculture & Veterinary Medicine, United Arab Emirates

**Keywords:** Strongyloides fuelleborni, Strongyloidiasis, Vervet, Saint Kitts, Genotyping, Population structure

## Abstract

Human strongyloidiasis is an important neglected tropical disease primarily caused by the nematode *Strongyloides stercoralis*, and to a lesser extent *Strongyloides fuelleborni* which mainly infects non-human primates. Zoonotic sources of infection have important implications for control and prevention of morbidity and mortality caused by strongyloidiasis. Recent molecular evidence suggests that for *S. fuelleborni,* primate host specificity is variable among genotypes across the Old World, and consequently that these types likely vary in their capacity for human spillover infections. Populations of free-roaming vervet monkeys (*Chlorocebus aethiops sabaeus*), introduced to the Caribbean Island of Staint Kitts from Africa, live in close contact with humans, and concern has arisen regarding their potential to serve as reservoirs of zoonotic infections. In this study, we sought to determine the genotypes of *S. fuelleborni* infecting St. Kitts vervets to explore whether they are potential reservoirs for human-infecting *S. fuelleborni* types. Fecal specimens were collected from St. Kitts vervets and *S. fuelleborni* infections were confirmed microscopically and by PCR. *Strongyloides fuelleborni* genotypes were determined from positive fecal specimens using an Illumina amplicon sequencing-based genotyping approach targeting the mitochondrial cox1 locus and 18S rDNA hypervariable regions I and IV of *Strongyloides* species. Phylogenetic analysis of resultant genotypes supported that *S. fuelleborni* from St. Kitts vervets is of an exclusively African variety, falling within the same monophyletic group as an isolate which has been detected previously in a naturally infected human from Guinea-Bissau. This observation highlights that St. Kitts vervets may serve as potential reservoirs for zoonotic *S. fuelleborni* infection, which warrants further exploration.

## Highlights

• Free-roaming vervets on the Caribbean Island of Saint Kitts are infected with *S. fuelleborni.*
• We genotyped *S. fuelleborni* from St. Kitts vervets to assess the zoonotic potential of these worms.
• St. Kitts vervets are infected with an African *S. fuelleborni* type that has been found previously in a human.
• St. Kitts vervets may therefore represent a reservoir of human *S. fuelleborni* infection.

## Graphical abstract

**Figure:**
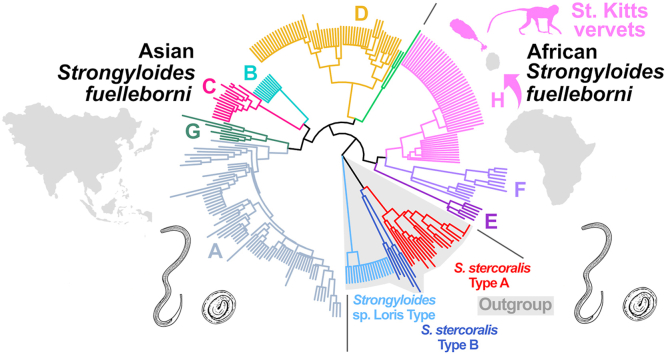

## 1 Introduction

Human strongyloidiasis is an important disease caused by the intestinal nematode *Strongyloides stercoralis*, and to a lesser extent *Strongyloides fuelleborni*. The clinical characteristics of strongyloidiasis are of variable severity, but generally include abdominal pain, diarrhea, skin rash, eosinophilia, and respiratory symptoms; disseminated strongyloidiasis or hyperinfections caused by *S. stercoralis* infection can be life-threatening because of autoinfection (Nutman, 2017). *Strongyloides* cycles between parasitic and free-living stages (Hunt et al., 2016), and the most common route of transmission is via direct penetration of exposed skin by larvae in contaminated soil. Infected human hosts will then pass larvae or eggs in the feces. Human strongyloidiasis is cosmopolitan in its distribution, and has an estimated global burden of approximately 600 million human infections (Nutman, 2017; Buonfrate et al., 2020). The largest burden of disease occurs in low socioeconomic contexts, due to the lack of access to clean water, sanitation and hygiene (i.e., WASH) interventions (Strunz et al., 2014; Garn et al., 2022).

Historically, *S. stercoralis* has been considered capable of infecting both humans and domestic dogs (*Canis lupus familiaris*), while *S. fuelleborni* has been recognized as a cause of strongyloidiasis in humans and non-human primates throughout the Old World (Barratt and Sapp, 2020). More recently, molecular evidence has revealed that multiple genetic subpopulations exist within *S. fuelleborni* and *S. stercoralis*, supporting that these two species may comprise species complexes (Barratt and Sapp, 2020). Two subspecies of *S. fuelleborni* are currently recognized; *S. fuelleborni fuelleborni* (*S. f. fuelleborni*) which has been reported in sub-Saharan Africa, South Asia, and southeast Asia (Barratt and Sapp, 2020), and *S. fuelleborni kellyi* (*S. f. kellyi*), which is an enigmatic species that possesses an ambiguous relationship with *S. f. fuelleborni* and is only found in Papua New Guinea (Dorris et al., 2002). *Strongyloides f. fuelleborni* infects Old-World primates, including pig-tailed macaques, Bornean orangutans, gorillas, chimpanzees, among others (Hasegawa et al., 2009, 2010, 2016; Kooriyama et al., 2012; Thanchomnang et al., 2017, 2018).

*Strongyloides fuelleborni* is recognized as a possible source of zoonotic strongyloidiasis via transmission from primates into nearby human populations or humans who encroach on the primates’ habitat. For Asian *S. fuelleborni* types, this spillover seems the most important route of infection, as documented cases involve people residing in close proximity to monkey troops harboring *S. fuelleborni*, or keeping monkeys as pets, in the absence of other likely exposures (Thanchomnang et al., 2018; Janwan et al., 2020). Anthroponotic transmission of Asian/southeast Asian types of *S. fuelleborni* is rare or absent. Regarding *S. fuelleborni* types from Africa, it is not known whether anthroponotic transmission is an important route of infection. However, prior analysis has shown close genetic clustering of some human-derived African *S. fuelleborni* types which might suggest that anthroponotic transmission occurs for certain African types (Barratt and Sapp, 2020). Among African varieties of *S. fuelleborni*, distinct genetic sub-structuring has been noted, with discrete clustering of Tanzanian types (East African) separately from Central and West African isolates, with human infections caused by both East and West African varieties (Hasegawa et al., 2010, 2016; Barratt and Sapp, 2020; Ko et al., 2023). Asian varieties also possess a genetic population substructure based on geography, with isolates from Borneo, mainland Southeast Asia, and Japan clustering discretely from one another (Barratt and Sapp, 2020; Ko et al., 2023).

African vervet monkeys (*Chlorocebus aethiops sabaeus*) exist as free-roaming populations on the Caribbean island of Saint Kitts, descended from a small group originally introduced by Europeans during the 17th century (McGuire, 1974). Since their introduction their numbers have exploded, and due to the damage they cause to a variety of crops/fruits, native flora, and wildlife, St. Kitts vervets are considered pests by local farming communities contrary to other government agencies who consider their presence on the Island an important tourist attraction. Recent surveys have shown that St. Kitts vervets are a host for multiple human-pathogenic helminths including *Trichuris trichiura*, *Schistosoma mansoni*, and *S. fuelleborni*, in addition to other protozoal, bacterial, and viral pathogens (Gallagher et al., 2019; Ketzis et al., 2020; Cruz et al., 2021). It is possible that vervets originally introduced to St. Kitts brought an African variety of *S. fuelleborni* to the island, although a detailed molecular analysis of *S. fuelleborni* types infecting St. Kitts vervets has not been performed. The large number of vervets on the island of St. Kitts and their curiosity causes them to regularly spill into human occupied areas of the island which could place the islands’ human inhabitants at risk of *S. fuelleborni* infection. Determining whether St. Kitts vervets are infected with *S. fuelleborni* types identified previously in humans might provide greater insight into the possible risk of zoonotic *S. fuelleborni* transmission on the island.

We sought to explore whether free-roaming vervets on the island of St. Kitts are reservoirs of *S. fuelleborni* types that have previously been reported to cause human strongyloidiasis. Using a next-generation sequencing (NGS)-based multi-locus-sequence-typing (MLST) method targeting the mitochondrial cox1 locus, and hypervariable regions (HVRs) –I and -IV of the 18S rDNA (Barratt et al., 2019a; Beknazarova et al., 2019), we genetically characterized *S. fuelleborni* from vervets inhabiting several regions of St. Kitts. Using a machine-learning-based genetic distance computation method to facilitate phylogenetic reconstructions (Barratt and Sapp, 2020; Jacobson et al., 2022), the genotypes were clustered alongside previously described *S. fuelleborni* genotypes from Africa and Asia, including types previously isolated from humans.

## 2 Materials and methods

### 2.1 Study population and specimen collection

The study population included multiple troops of free-roaming African vervet monkeys (*Chlorocebus aethiops sabaeus*) from the island of Saint Kitts. Fecal specimens were collected from the transport cages of vervets trapped for other purposes (relocating, contract research) and stored at 4 °C until an initial screening (direct smear with Lugol's iodine; within 3–5 days of collection). Samples identified with *Strongyloides* in this initial screen (n = 34) were placed directly into 90% ethanol (1 g–3 mL) and stored at −20 °C until shipment to the Parasitic Diseases Branch (PDB) at the US Centers for Disease Control and Prevention (CDC) for all subsequent analyses, including *S. fuelleborni* genotyping, and a secondary microscopic screen (400× magnification) of wet mounts for the presence of *Strongyloides* eggs and/or larvae. The specimens were stored at 4 °C prior to subsequent molecular analyses. A single bronchoalveolar lavage (BAL) fluid specimen from a human patient who tested positive for *S. stercoralis* in the parasitology reference laboratory at CDC (by PCR and microscopy) was included in this study as a positive control*.*

### 2.2 Ethics

The collection of fecal specimens from St. Kitts vervets was approved by the Ross University School of Veterinary Medicine Institutional Animal Care and Use Committee, Tissue/Sample Use application #TSU10.23.19. Ethics approval for the use of anonymized, de-identified, human samples as non-engaged research was granted by the CDC Center for Global Health Human Subjects Review, approval number 2016-314.

### 2.3 Extraction of DNA from vervet fecal specimens

DNA was extracted from vervet fecal specimens using a DNeasy PowerSoil Kit (Qiagen, Germantown, Maryland, USA) with some minor modifications. As the fecal specimens had been preserved in 70% ethanol, they were washed in PBS prior to DNA extraction. To do this, approximately 0.25 g of feces suspended in ethanol was placed in a 2 mL Eppendorf tube and centrifuged in a benchtop centrifuge (Eppendorf Centrifuge 5424) at maximum speed (15,000 RPM). The supernatant was discarded. Next, the tube was filled to 2 mL with PBS and vortexed to disrupt the pellet. The suspension was centrifuged at maximum speed once more and the supernatant was removed again. The resulting pellet was transferred to a PowerBead Tube and subjected to Bead beating for 3 min at 1400 RPM on an Eppendorf Thermomixer. After adding solution C1 to the tube containing the PowerBeads and feces, we also added 20 mL of proteinase K taken from a DNEasy Blood & Tissue Kit (Qiagen). The solution was gently vortexed and left to digest at 65 °C for 30 min. The digested specimen was vortexed again following digestion. The DNA was eluted in a volume of 50 μL of Solution C6.

### 2.4 Polymerase chain reaction

Three genotyping loci were amplified from each specimen by PCR; Cytochrome *c* oxidase subunit 1 (cox1), 18S rDNA hypervariable region I (HVR-I), and 18S rDNA hypervariable region IV (HVR-IV). The primer sequences used to amplify each locus are provided in Table 1. Amplification reactions were prepared to contain 2.5 μL of each forward and reverse primer (10 μM solutions), 18 μL of PCR grade water, 25 μL of NEB Next Q5 Hot Start HiFi PCR Master Mix (New England Biolabs, Ipswich, MA, USA), and 2 μL of template DNA. For cox1 and HVR-I, temperature cycling conditions were as follows; initial melting at 98 °C for 2 min followed by 45 cycles of 98 °C for 10 s, annealing at 65 °C for 10 s, and extension 72 °C for 10 s. The protocol concluded with a final extension of 72 °C for 2 min, and holding at 4 °C. For HVR-IV, temperature cycling conditions were as follows: initial melting at 98 °C for 2 min followed by 45 cycles of 98 °C for 10 s, annealing at 63 °C for 10 s, and extension 72 °C for 10 s. The protocol concluded with a final extension step of 72 °C for 2 min, and holding at 4 °C. All PCR runs were accompanied by a negative template control (PCR grade water added to a reaction in place of DNA), and a positive template control comprising DNA extracted from a bronchoalveolar lavage (BAL) fluid specimen that had previously tested positive by PCR and microscopy for *S. stercoralis* in the diagnostic parasitology reference laboratory at CDC*.*

**Table 1:** PCR primers and reaction conditions.

Target locus	Primer	Amplicon Length ^α^	Sequence	Annealing temperature
COXI	SSP_COX1_F*	217 bp	5′-TTTGATCCTAGTTCTGGTGGTAATCC-3′	65 °C
	SSP_COX1_R*		5′-GTAGCAGCAGTAAAATAAGCACGAGA-3′	
18S rDNA HVR-I	NEW_HVR_I_F	∼434 bp ^β^	5′-GCTCATTATAACAGCTATAGACTACACGGTA-3′	65 °C
	NEW_HVR_I_R		5′-CCACAACAATCATTTTATGCACTTGG-3′	
18S rDNA HVR-IV	NEW_HVR_IV_F	∼255 bp ^β^	5′-CGGGCCGGACACTATAAGG-3′	63 °C
	NEW_HVR_IV_R		5′-ATCTCTAAACAGGAACATAATGATCACTAC-3′	

^α^ Excluding PCR primer sequences from the amplicon.

^β^ Length varies depending on genotype.

^∗^ Broadly specific primers for generation of *Strongyloides* sp. *cox*1 amplicons as well as those of several strongyles.

### 2.5 Illumina amplicon sequencing and bioinformatic analysis

The sequencing methods employed here were modified from those described by Beknazarova et al. (2019) and Barratt et al. (2019a). Briefly, each amplicon was separately purified and normalized using a SequalPrep Normalization Kit (Thermo Fisher Scientific Waltham, Massachusetts, USA) with an elution volume of 20 μL. After purification and normalization, equal volumes of each amplicon from each fecal specimen were pooled and the resulting normalized pool of amplicons was subjected to library preparation using Nextera XT DNA Library Prep Kit in accordance with the manufacturer's instructions (Illumina, San Diego, California, USA). Next, DNA concentration and DNA molecular size for the resultant Illumina library were assessed using a Qubit dsDNA HS Assay Kit (Thermo Fisher Scientific) and a High Sensitivity D1000 ScreenTape on the 2200 TapeStation (Agilent, Santa Clara, California, USA), respectively. The quantified library was diluted to 10–15 pM and sequenced on the MiSeq Platform using the MiSeq Reagent Kits V2 (500 cycle) (Illumina). Analysis of Illumina data was performed using a custom Geneious workflow (Geneious Prime, version 2022: www.geneious.com). Briefly, this workflow performed read quality control and identification of the haplotype composition of each specimen at the three loci sequenced. This workflow is described in detail in the studies by Beknazarova et al. (2019) and Barratt et al. (2019a). A detailed description of the haplotype naming conventions used in this study is provided in later sections.

### 2.6 Reference dataset of published *Strongyloides* genotypes

We used 128 *S. fuelleborni* genotypes compiled as part of previous work (Barratt et al., 2019a; Barratt and Sapp, 2020), plus 18 from an undefined *Strongyloides* species identified in Bornean slow lorises (Frias et al., 2018). Our dataset included 24 cox1 sequences and HVR-IV sequences from Thai isolates of *S. fuelleborni*: one from a human and 23 from pig-tailed macaques (*Macaca nemestrina*) (Janwan et al., 2020). We also included 39 *S. fuelleborni* cox1 sequences generated by Ko et al. (2023) from a range of Asian non-human primates including rhesus macaques from Myanmar (*Macaca mulatta*), Japanese macaques (*Macaca fuscata*), and sequences from four primate species housed in zoological parks in Japan; siamang (*Symphalangus syndactylus*), red-shanked douc (*Pygathrix nemaeus*), Francois’ langur (*Trachypithecus francoisi*), and Bornean Orangutan (*Pongo pygmaeus*) (Ko et al., 2023). To serve as an outgroup for our phylogenetic analysis, we sequenced our *S. stercoralis* PCR positive control amplicons in the same Illumina library as the vervet samples, and included 28 previously published *S. stercoralis* genotypes (Barratt and Sapp, 2020), plus a cox1 sequence extracted from the mitochondrial genome of *S. stercoralis* reference strain PV001 (GenBank [GB] - accession: NC_028624.1). A detailed overview of all specimens within this dataset, including information on hosts, genotype, and GenBank accession numbers, is provided in File S1, Tab A.

### 2.7 Assigning haplotype names for construction of genotypes

To characterize the genotypes from our reference isolates (and from the St. Kitts vervets), all cox1, 18S HVR-I, and HVR-IV sequences were assigned a haplotype name following earlier haplotype naming conventions developed for *Strongyloides* sp. (Jaleta et al., 2017; Barratt et al., 2019a; Barratt and Sapp, 2020), with some modifications for cox1 (discussed in a later section). Haplotype names were assigned by BLASTN comparison against the fasta sequences provided in File S2. These BLASTN searches were executed using the Geneious Prime interface, requiring hits of 100% sequence identity. BLASTN results were exported from Geneious in text format, where each text file contained the list of haplotype names detected in a given isolate. These text files were used to construct a haplotype data sheet (HDS); a condensed format for representing haplotype data (File S1, Tab B and Tab D) which is the required input format for computation of genetic distances using Barratt's heuristic (Barratt et al., 2021; Jacobson et al., 2022) – see https://github.com/Joel-Barratt/Eukaryotyping. In all, the HDS contained 30 genotypes from *S. stercoralis* and 18 genotypes from the loris-derived *Strongyloides* sp., plus 191 published *S. fuelleborni* genotypes, and 48 *S. fuelleborni* genotypes generated here from St Kitts vervets (see results).

### 2.8 Establishing minimum data requirements and cox1 haplotype definitions

Barratt's heuristic was used to compute pairwise genetic distances for phylogenetic tree construction (Barratt et al., 2019b; Nascimento et al., 2020; Jacobson et al., 2022). Barratt's heuristic was used because it can compute distances for datasets comprising isolates sequenced at different but overlapping combinations of markers. Many of the genotypes examined here were sequenced as part of separate, unrelated studies, so the markers sequenced were not always the same. Barratt's heuristic accommodates such datasets by imputing missing distance values when comparing isolates that have not been sequenced at the same loci (Jacobson et al., 2022). This method was designed for large datasets, and as the number of isolates with mismatched markers increases within a dataset, and/or as the number of shared loci sequenced between a given isolate pair decreases, the more tenuous these imputations become (Barratt and Sapp, 2020; Jacobson et al., 2022). Consequently, realistic minimum data requirements and maximum limits on the number of markers for which imputation is attempted must be established prior to analysis (Jacobson et al., 2022). A detailed description of how these minimum data requirements and maximum imputation limits were established, is provided in Supplementary File S1, Appendix A.

### 2.9 Distance computation and construction of phylogenetic trees

Genetic distance computation was performed using Barratt's heuristic via the scripts and instructions available here: https://github.com/Joel-Barratt/Eukaryotyping. Our final calibrated HDS was used as input for distance computation and subsequent tree construction. The resultant pairwise matrix was clustered via the ‘agnes’ R package using Ward's method (Ward, 1963) to generate a hierarchical tree. We also generated a neighbor-joining tree (Saitou and Nei, 1987) from the same distance matrix using the ‘nj’ function available in the ‘ape’ R package. The ‘root’ function in the ‘ape’ R package was used to root the tree at the *S. stercoralis*/*Strongyloides* sp. ‘Loris’ clade – establishing this clade as the outgroup. The ‘ggtree’ R package was used to visualize and annotate the resultant trees. Images of relevant hosts were obtained from PhyloPic (http://phylopic.org) or prepared in-house for annotation of dendrograms. Maps were generated in R using ggplot. Images were rendered using the GNU Image manipulation program (https://www.gimp.org).

## 3 Results

### 3.1 Morphological examination and amplicon sequencing

**Fig. 1:**
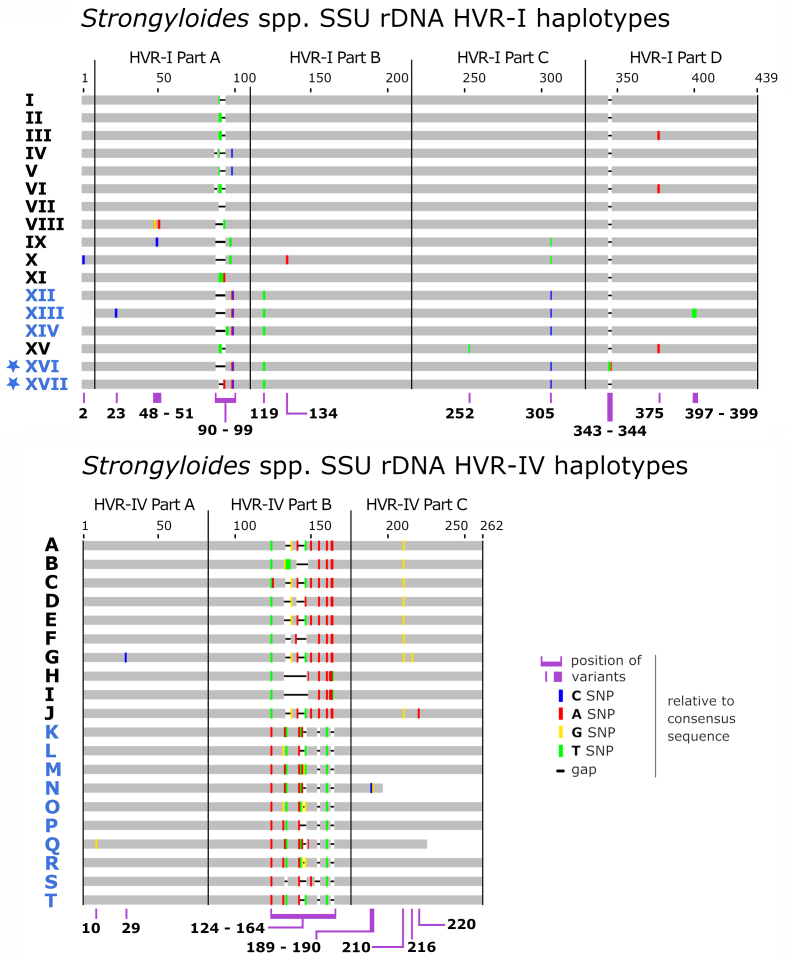
Schematic of the *Strongyloides* genotyping scheme referenced here Graphical representation a *Strongyloides* sp. genotyping scheme after the description of Barratt et al. (Barratt et al., 2019a; Barratt and Sapp, 2020). This scheme was expanded here to include haplotypes XVI and XVII of 18S HVR-I (indicated by a star) identified here from feral vervet monkeys living on the island of St Kitts (GenBank accessions in Table 3). Haplotype names shown in blue belong to *S. fuelleborni* and those shown in black belong to other *Strongyloides* species in accordance with this typing scheme. Haplotype sequences are provided in File S1. (For interpretation of the references to color in this figure legend, the reader is referred to the Web version of this article.)

Larvated eggs consistent with *Strongyloides* sp. were observed microscopically in 29 of the 34 fecal specimens during the secondary microscopic screening performed at CDC (Table 2). HVR-I and HVR-IV sequences were obtained from all 34 specimens, while a cox1 sequence was not obtained for 6 of these. Between one and three *Strongyloides* sp. cox1 sequences of 217 bp were detected in these 28 specimens. Haplotype XII of HVR-I, which is consistent with *S. fuelleborni* from African primates (Table 3), was detected in all 34 specimens. Additionally, two novel HVR-I haplotypes (XVI and XVII) were detected. These two sequences were confirmed as belonging to *S. fuelleborni* based on BLASTN searches against the NCBI nucleotide database where the nearest matches (∼99.5% identity) included hits to *S. fuelleborni* 18S sequences. Haplotypes XVI and XVII possessed a ‘T’ SNP at position 119, and a ‘C’ SNP at position 305 relative to the alignment in Fig. 1; these SNP's are characteristic of all known HVR-I sequences from *S. fuelleborni*. The HVR-IV haplotypes L, T, M, O, R, and P detected in these samples are also characteristic of *S. fuelleborni* from African primates (Table 3).

**Table 2:** Results of morphological screen for *Strongyloides* sp. forms from trapped vervets†

Animal ID	Specimen number	Trapping location	*Strongyloides* sp. (eggs)	*Strongyloides*-like larvae*	Cox1 (# of sequences)∗∗	HVR-I (haplotypes detected)∗∗∗	HVR-IV (haplotypes detected)∗∗∗
			CDC morphological screen		PCR and sequencing results (positive: + or negative:)		
9808	Specimen 1	Cayon	**+**	**-**	+ (2)^c^^,^^f^	XII, XVI, XVII	T, M, O R, P
9772	Specimen 2	Not recorded‡	**+**	**-**	+ (1)^f^	XII, XVI, XVII	L, T, M, O, R, P
9809	Specimen 3	Cayon	**+**	**-**	–	XII, XVI, XVII	L, T, M, O, R, P
9749	Specimen 4	Not recorded‡	**+**	**-**	–	XII, XVI, XVII	L, T, M, R, P
9767	Specimen 5	Not recorded‡	**+**	**-**	+ (3)^a^^,^^e^^,^^f^	XII, XVI, XVII	L, T, M, R, P
9862	Specimen 6	Phillips	**+**	**-**	–	XII, XVI, XVII	L, T, M, R, P
9852	Specimen 7	Dieppe Bay	**-**	**-**	–	XII, XVI, XVII	L, T, M, R, P
9855	Specimen 8	Cedar Grove	**+**	**-**	+ (1)^f^	XII, XVI, XVII	L, T, M, R, P
9859	Specimen 9	Cedar Grove	**+**	**-**	–	XII, XVI, XVII	L, T, M, O, R, P
9861	Specimen 10	Cedar Grove	**+**	**-**	–	XII, XVI, XVII	L, T, M, O, R, P
9858	Specimen 11	Cedar Grove	**+**	**-**	+ (1)^f^	XII, XVI, XVII	L, T, M, R, P
9863	Specimen 12	Phillips	**-**	**-**	+ (1)^f^	XII, XVI, XVII	L, T, M, R, P
9788	Specimen 13	Cedar Grove	**+**	**-**	+ (2)^d^^,^^f^	XII, XVI, XVII	L, T, M, R, P
9786	Specimen 14	Cedar Grove	**-**	**-**	+ (1)^f^	XII, XVI, XVII	L, T, M, R, P
9868	Specimen 15	Cedar Grove	**+**	**-**	+ (2)^d^^,^^f^	XII, XVI, XVII	L, T, M, R, P
9873	Specimen 16	Cedar Grove	**+**	**-**	+ (1)^f^	XII, XVI, XVII	L, T, M, R, P
9875	Specimen 17	Monkey Hill	**+**	**-**	+ (3)^d^^,^^e^^,^^f^	XII, XVI, XVII	L, T, M, R, P
9876	Specimen 18	Cedar Grove	**+**	**-**	+ (1)^f^	XII, XVI, XVII	L, T, M, R, P
9867	Specimen 19	Cedar Grove	**+**	**-**	+ (2)^a^^,^^f^	XII, XVI, XVII	L, T, M, R, P
9871	Specimen 20	Cedar Grove	**+**	**-**	+ (2)^a^^,^^f^	XII, XVI, XVII	L, T, M, R, P
9866	Specimen 21	Cedar Grove	**+**	**-**	+ (1)^f^	XII, XVI, XVII	L, T, M, O, R, P
9893	Specimen 22	Cedar Grove	**+**	**-**	+ (1)^f^	XII, XVI, XVII	L, T, M, R, P
9894	Specimen 23	Cedar Grove	**+**	**+**	+ (2)^e^^,^^f^	XII, XVI, XVII	L, T, M, R, P
9896	Specimen 24	Cedar Grove	**-**	**-**	+ (1)^f^	XII, XVI, XVII	L, T, M, R, P
9898	Specimen 25	Cedar Grove	**+**	**-**	+ (3)^a^^,^^b^^,^^f^	XII, XVI, XVII	L, T, M, R, P
9897	Specimen 26	Cedar Grove	**+**	**-**	+ (3)^a^^,^^e^^,^^f^	XII, XVI, XVII	L, T, M, O, R, P
9750	Specimen 27	Not recorded‡	**+**	**+**	+ (1)^f^	XII, XVI, XVII	L, T, M, R, P
9758	Specimen 28	Not recorded‡	**+**	**-**	+ (1)^f^	XII, XVI, XVII	L, T, M, O, R, P
9845	Specimen 29	Dale Mountain	**-**	**-**	+ (2)^e^^,^^f^	XII, XVI, XVII	L, T, M, R, P
9853	Specimen 30	Sir Gillian, Old Road	**+**	**-**	+ (3)^a^^,^^d^^,^^f^	XII, XVI, XVII	L, T, M, R, P
9844	Specimen 31	Dale Mountain	**+**	**-**	+ (2)^a^^,^^f^	XII, XVI, XVII	L, T, M, O, R, P
9842	Specimen 32	Cedar Grove	**+**	**-**	+ (1)^f^	XII, XVI, XVII	L, T, M, R, P
9850	Specimen 33	Lodge	**+**	**-**	+ (1)^f^	XII, XVI, XVII	L, T, M, O, R, P
9849	Specimen 34	Lodge	**+**	**+**	+ (3)^a^^,^^e^^,^^f^	XII, XVI, XVII	L, T, M, O, R, P

Notes: HVR-I haplotypes XVI and XVII are novel sequences detected for the first time in this study. Additionally, please note that all sequencing results were obtained from individual fecal samples from individual monkeys (not pooled samples). Therefore, individual vervets harbored multiple genotypes of *S. fuelleborni*.

† These are the results obtained for the secondary microscopic screen performed at CDC after specimens were received there and following the initial screen performed on St. Kitts shortly after specimen collection.

‡ The vervets that produced these specimens are thought to have been trapped at Greenhill, though this is uncertain.

∗∗ The precise Cox1 sequence/s detected in each specimen can be determined using the footnotes (a-f) below, where GenBank accession numbers (GB) are listed for each Cox1 sequence.

a Type 1 - GB: OQ190184.

b Type 2 - GB: OQ190185.

c Type 3 - GB: OQ190186.

d Type 4 - GB: OQ190187.

e Type 5 - GB: OQ190188.

f Type 6 - GB: OQ190189.

∗∗∗ See Table 3 to obtain GenBank accession numbers for HVR sequences.

**Table 3:** HVR-I and HVR-IV haplotypes of *Strongyloides fuelleborni*.

Haplotypes	GenBank Accession/s	Hosts
HVR-I haplotypes of *S. fuelleborni*		
XII	AB453320.1, AB453321.1, AB821045.1, AB821046.1, MN076377 – MN076380, OQ190413	gorillas, chimpanzees, humans, baboons, vervets∗∗
XIII	AB453322.1	Gorilla
XIV	AB677955.1, AB272235.1, AB453317.1, AB453318.1, AB453319.1	Japanese macaque
XVI∗	OQ190414	vervets
XVII∗	OQ190415	vervets
**HVR-IV haplotypes of *S. fuelleborni***		
K	LC085496.1, LC085493.1, LC085492.1, LC085491.1, LC085484.1, LC085494.1, AB526820.1, AB453320.1, AB526823.1, AB526821.1	chimpanzees and human
L∗∗	LC085490.1, LC085489.1, OQ190398	gorilla, vervets
M∗∗	LC085486.1, MN076407, OQ190400	human, vervets
N	LC085497.1	Chimpanzee
O∗∗	AB453322.1, OQ190401	gorilla, vervets
P∗∗	LC085488.1, AB526824.1, AB526825.1, OQ190402	chimpanzee, gorilla, vervets
Q	AB526822.1	baboon
R∗∗	AB453321.1, OQ190403	chimpanzee, vervets
S	KY081222.1, AB453317.1, AB453318.1, AB453319.1, AB272235.1, MH045486.1, MH045487.1, MH045487.1, MN076415, MT484268, MT484269	human, long-tailed macaque, pig-tailed macaque
T∗∗	MN076408, OQ190399	human, vervets

Note: haplotype XV of HVR-I was identified in an earlier study (Barratt and Sapp, 2020) and is intentionally excluded from this table as that haplotype belongs to *S. stercoralis* (table only shows *S. fuelleborni* haplotypes).

∗ Novel haplotypes detected for the first time in the present study in African vervets on St Kitts.

∗∗ Haplotypes that have been identified in previous studies from primates in Africa that were also identified here in African vervets from St. Kitts.

### 3.2 *Constructing* S. fuelleborni *genotypes using NGS data from St Kitts vervets*

The *S. fuelleborni* genotypes obtained from the vervets were mixed, where three HVR-I haplotypes (XII, XVI, XVII), and 5 or 6 HVR-IV haplotypes (L, T, M, R, P or L, T, M, O, R, P) were detected in all specimens (Table 2). Therefore, it was not possible to determining the HVR-I and HVR-IV haplotype combinations that belonged to a given cox1 sequence to construct individual underlying genotypes. However, segments B and C of HVR-I are identical for haplotypes XII, XVI and XVII (Fig. 1). Additionally, segments A and C are identical across haplotypes L, T, M, O, R and P (Fig. 1). Subsequently, to maximize genotype completeness for distance computation, all cox1 sequences from vervets were linked to HVR-I segments B and C, plus HVR-IV segments A and C, when constructing genotypes (i.e., haplotype lists) for entry into the HDS. In all, 48 *S. fuelleborni* cox1 sequences of 217 bp (i.e., segments J3 through O1) were generated from St. Kitts vervets and each was used to construct a single genotype, including the cox1 sequence and the conserved segments of HVR-I and HVR-IV.

### 3.3 Phylogenetic analysis

**Fig. 2:**
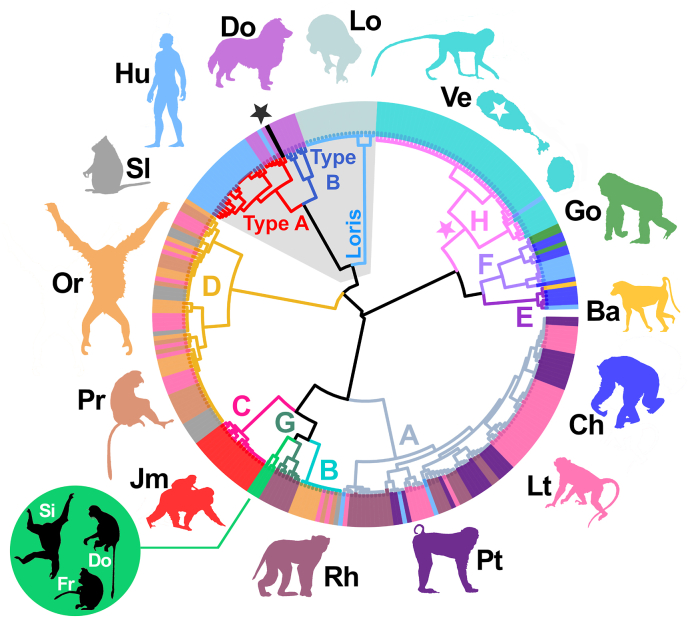
Hierarchical tree of clustered distances generated from genotyped *S. fuelleborni* from St Kitts vervets and other *Strongyloides* sp. genotypes This unrooted tree was generated using Wards method (Ward, 1963) to cluster a pairwise distance matrix computed from 285 *Strongyloides* genotypes, including 48 from St. Kitts vervets. Branches are colored according to their cluster membership (A through H). We introduce *S. fuelleborni* type H (pink star) identified from St. Kitts vervets, noting that an H-type isolate was found previously in a human from Guinea-Bissau. Colored peripheral bars reflect the host species from which isolates were derived; dog (Do), human (Hu), chimpanzee (Ch), lorises (Lo), long-tailed macaques (Lt), pig-tailed macaques (Pt), Japanese macaques (Jm), proboscis monkeys (Pr), silvered leaf monkeys (Sl), orangutans (Or), Rhesus macaques (Rh), St Kitts (white star) vervets (Ve), gorilla (Go), and baboon (Ba). Divergent *S. fuelleborni* types described by Ko et al. (2023) were also clustered (green circle and branches) from Siamang (Si), Douc (Do), and Francois' langur (Fr) housed in zoological parks in Japan. The black bar and black star indicate *S. stercoralis* reference strain PV001. *Strongyloides stercoralis* types A and B, are shown with red and blue branches respectively. The loris clade is shown in light blue. The *S. stercoralis* and loris clades clustered here for comparison are shaded in a gray background. A version of this same tree with isolate names shown on the branch tips is provided in File S3; Tree A. (For interpretation of the references to color in this figure legend, the reader is referred to the Web version of this article.)

**Fig. 3:**
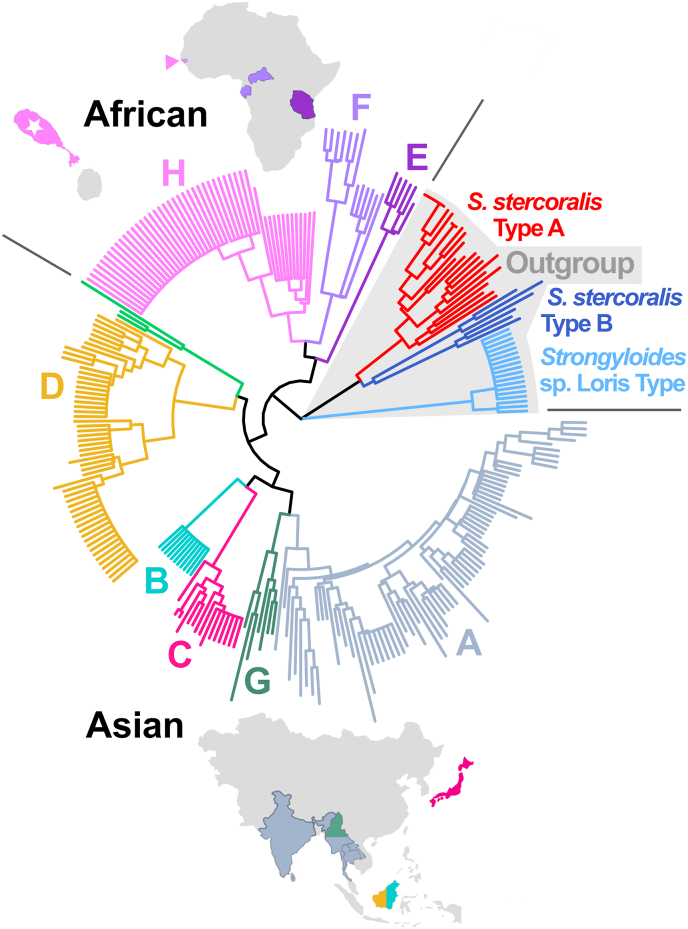
Neighbor-Joining tree generated from St Kitts *S. fuelleborni* genotypes and other *Strongyloides* types This tree was generated by applying the Neighbor-Joining clustering method (Saitou and Nei, 1987) to a pairwise distance matrix computed using Barratt's heuristic from 285 *Strongyloides* genotypes, including 48 from St. Kitts vervets. Branches are colored according to their cluster membership (A through H) as defined by Ko et al. (2022). We also introduce *S. fuelleborni* type H (light pink) from St. Kitts vervets, noting that an H-type isolate was found previously in a human from Guinea-Bissau (pink triangle). Divergent *S. fuelleborni* types described by Ko et al. (2022) were also analyzed (bright green branches without a cluster letter) from Siamang, Douc, and Francois' langur housed in zoological parks in Japan. A version of this same tree with isolate names shown on the branch tips is provided in File S3; Tree B. (For interpretation of the references to color in this figure legend, the reader is referred to the Web version of this article.)

Two of the 287 genotypes failed to meet the minimum data requirements for distance computation (fewer than 14 cox1 segments) and were excluded, leaving 285 genotypes for phylogenetic analysis. A robust phylogenetic reconstruction was achieved by analyzing any HVR data available for these 285 types plus cox1 segments I1 through O1 (i.e., 19 segments, or 285 bp), which constitutes an additional five cox1 segments beyond the 14 segments defined within the 217 bp amplicon (see HDS, File S2, Tab B). The pairwise distance matrix (File S2, Tab C) generated from this HDS was clustered using Wards method to construct a hierarchical tree (Fig. 2), and using the Neighbor-Joining method to produce a phylogeny (Fig. 3). These trees supported that the 48 *S. fuelleborni* genotypes from St. Kitts vervets were of an African variety, forming a unique cluster within the larger monophyletic African clade. This African clade includes *S. fuelleborni* types causing infections in chimpanzees, gorillas, baboons, and humans (Figs. 1 and 2). Additionally, an *S. fuelleborni* genotype sequenced previously from a human in Guinea-Bissau (Barratt et al., 2019a) clustered within the St. Kitts group (cluster H, Fig. 2). The Neighbor-Joining tree revealed a population structure that was consistent with that presented by Ko et al. (2023), which included many of the same reference isolates. As such, these trees were annotated (Figs. 2 and 3) following the convention of Ko et al. (2023) who introduced *S. fuelleborni* types A through G. Cluster H (introduced here) includes *S. fuelleborni* from St. Kitts vervets and the type detected in a human in Guinea-Bissau.

## 4 Discussion

We genetically characterized *S. fuelleborni* from St. Kitts vervets and confirmed that they are genetically similar to a *S. fuelleborni* type found previously in a human from Guinea-Bissau (Barratt et al., 2019a). This supports that *S. fuelleborni* types infecting St. Kitts vervets were brought to the island with the small group of vervets originally introduced by Europeans from Africa during the 17th century (McGuire, 1974). Clustering of *S. fuelleborni* genotypes from St. Kitts alongside genotypes of other *S. fuelleborni* varieties demonstrated that St. Kitts *S. fuelleborni*, in addition to the isolate from Guinea-Bissau, represent a novel variety which we designate as *S. fuelleborni* Type H, following the convention of Ko et al. (2023).

As *S. fuelleborni* Type H was identified here in St. Kitts vervets and previously in a naturally infected human, it is possible that these vervets may serve as a reservoir for human infection via zoonotic spillover as vervet populations encroach on human-occupied areas of the island. While human infections with *S. stercoralis* on St. Kitts have been documented (Ketzis and Conan, 2017), data on *S. fuelleborni* infections in the human inhabitants of St. Kitts are lacking. However, *S. fuelleborni* infections in humans are likely underreported globally, due to misidentification of the eggs as those of other nematodes and because the eggs are typically shed in low numbers (Potters et al., 2020). Additionally, rhabditiform larvae may hatch from eggs in the stool if processing is delayed, and the likely outcome of this would be a misidentification as *S. stercoralis* larvae (which are morphologically identical to those of *S. fuelleborni*) (Ashford and Barnish, 1989). Therefore, the possibility remains that human infections are occurring or could occur on the island, and isolates from humans in St. Kitts are required to investigate this hypothesis. On the other hand, our data support that St. Kitts vervets are unlikely to be important reservoirs of *S. stercoralis* infection; given the sensitive deep amplicon sequencing methodology employed, it seems possible that low-level coinfections with *S. stercoralis* may have been encountered if this were the case, though we observed no evidence for this in the present study.

The findings of Ko et al. (2023) are mirrored here, indicating that globally distributed *S. fuelleborni* populations display genetic population sub-structuring based largely on geography, with Asian and African varieties being monophyletic. Sub-structuring based on the distribution of specific hosts seems of lesser importance versus geographic origin, suggestive of allopatric divergence. *Strongyloides fuelleborni* includes a diverse array of geographically and genetically distinct subpopulations (A through H), lending further support to the hypothesis that *S. fuelleborni* is not a single species but rather exists as a species complex as previously proposed (Barratt and Sapp, 2020). Within these subpopulations, the proportion of human infections appears to vary from rare, such as in *S. fuelleborni* complex type A to an unusual sub-cluster of *S. fuelleborni* complex type F which was isolated exclusively from humans. Studies involving broad sampling of both human and non-human primate hosts within regions known to be *S. fuelleborni*-endemic will be necessary to better understand the predilection for zoonotic versus anthroponotic transmission within the *S. fuelleborni* complex.

Our choice of Barratt's heuristic for genetic distance computation is noteworthy and has proven useful for investigating relationships among complex groups of sexually reproducing parasites (Jacobson et al., 2022). This approach was first applied to a *Strongyloides* MLST dataset by Barratt and Sapp (2020) to maximize the variety of *Strongyloides* types (and the number of loci) that could be included in a clustering meta-analysis, despite that the combinations of loci sequenced varied between studies included in this meta-analysis (Sato et al., 2007; Hasegawa et al., 2009, 2010, 2016; Makouloutou et al., 2014; Huong Giang et al., 2017; Thanchomnang et al., 2017, 2018; Barratt et al., 2019a; Janwan et al., 2020). As discussed above, Barratt's heuristic implements routines that impute distance values for each missing marker when comparing isolates sequenced at different but overlapping marker combinations (Jacobson et al., 2022). It can also compute distances between isolates possessing heterozygosity at one or more loci, where all alleles contribute to resultant tree structures (Jacobson et al., 2022). Despite its unique benefits, Barratt's heuristic possesses some important limitations that are best understood in the context of the rationale underpinning the method, which includes steps that scale dissimilarity scores generated for each locus by frequentist probabilities and entropy (Nascimento et al., 2020). Consequently, this method is best suited to massive datasets where isolates number in the hundreds to thousands (Barratt and Sapp, 2020; Nascimento et al., 2020). This is because the method assumes a wide, representative sampling to help ensure that isolates being analyzed are more likely to represent the diversity of isolates in nature. In line with this, the present study includes numerous isolates of *S. fuelleborni* from a broad range of geographic locations.

Though Barratt's heuristic allows comparison of sequences with missing data, for accurate imputation, at least some isolates should possess data for all segments of the loci being considered. As detailed above, the imputation steps have limits and attempting to impute values beyond these limits can lead to tenuous tree structures. Therefore, it was necessary to systematically reduce the ‘frame’ of cox1 considered for distance computation to avoid exceeding these imputation limits, which eventually yielded a tree that conformed well to established relationships between key *S. fuelleborni* varieties included in our reference population (Jaleta et al., 2017; Frias et al., 2018; Janwan et al., 2020; Ko et al., 2023). Calibration of our analysis based on established relationships previously observed among our reference genotypes ensures that our novel *S. fuelleborni* type H would be placed accurately within our final phylogenetic tree structure.

The prior studies by Ko et al. (2023) and Janwan et al. (2020) present Neighbor-Joining haplotype networks generated by aligning a 710 bp fragment of cox1 from all isolates. As haplotype network analysis (and other alignment-based phylogenetic techniques) requires isolates to be sequenced at precisely the same loci (Jacobson et al., 2022), performing a similar analysis that includes the same isolates analyzed here would require trimming of all cox1 sequences to 217 bp, and exclusion of all HVR data. As our MLST method was designed specifically for adaptation to the Illumina MiSeq platform, the cox1 amplicons generated are intentionally short to ensure they would fit within the span of a single paired-end Illumina read (Barratt et al., 2019a; Beknazarova et al., 2019). Using Barratt's heuristic in this study facilitated a phylogenetic meta-analysis that included almost all *S. fuelleborni* MLST genotypes currently available in GenBank despite inconsistencies in the loci (including the region of cox1) sequenced between studies, highlighting the utility and potential benefits of this method.

Our genetic characterization of *S. fuelleborni* from St. Kitts vervets sheds further light on the global population structure of the *S. fuelleborni* complex while also revealing preliminary evidence of zoonotic risk. Our phylogenetic reconstructions conformed with prescient knowledge of the global population structure of the *S. fuelleborni* complex. Though reconstruction of complete genotypes based on Illumina sequenced PCR products from fecal DNA was not possible, our data successfully confirmed the African origin of *S. fuelleborni* from St Kitts and was sufficient to identify *S. fuelleborni* type H as a previously unrecognized sub-population of the *S. fuelleborni* complex. Notably, a member of *S. fuelleborni* type H was observed previously in a human from Guinea-Bissau, highlighting the potential for zoonotic spillover of *S. fuelleborni* from St. Kitts vervets. Surveillance for human infections on St. Kitts and genotyping of isolates will help to further elucidate the role of vervets as potential reservoirs of human *S. fuelleborni* infection on the island. Overall, these data add to the growing understanding of *S. fuelleborni* as a species complex with more nuanced geographic and host relationships than what was assumed prior to the molecular era.

## Disclaimer

The findings and conclusions in this manuscript are those of the authors and do not necessarily represent the official position of the Centers for Disease Control and Prevention/the Agency for Toxic Substances and Disease Registry.

## Author contributions

**JB:** Conception, study design, PCR, sequencing, data analysis, bioinformatics, data interpretation, manuscript editing. **TR:** Study design, PCR, sequencing, drafting of manuscript. **JKK:** Collection and screening of samples, review of manuscript **SM:** Conception and review of manuscript. **SS:** microscopy, manuscript editing. **YQ:** study coordination and funding, review of manuscript.

## Declaration of competing interest

The authors of this manuscript have no conflicts of interest to disclose.
