# Supplementary material for: Genetic characterization of Strongyloides fuelleborni infecting free-roaming African vervets (Chlorocebus aethiops sabaeus) on the Caribbean island of St. Kitts
Source: Int J Parasitol Parasites Wildl. 2023 Feb 16;20:153–61. doi: 10.1016/j.ijppaw.2023.02.003 (PMC9969202; doi:10.1016/j.ijppaw.2023.02.003)
Supplement: Multimedia component 1 [file mmc1.docx]

**Supplemental File S2 for:**

Genetic characterization of *Strongyloides fuelleborni* infecting free-roaming African vervets (*Chlorocebus aethiops sabaeus*) on the Caribbean island of St. Kitts

Travis Richins^1,2^, Sarah Sapp^2^, Jennifer K. Ketzis^3^, Arve Lee Willingham^4^, Samson Mukaratirwa^3^, Yvonne Qvarnstrom^1^, Joel L. N. Barratt^1^*

^1^ Centers for Disease Control and Prevention, Division of Parasitic Diseases and Malaria, Parasitic Diseases Branch

^2^ Oak Ridge Associated Universities, Oak Ridge, Tennessee

^3^Biomedical Sciences, One Health Center for Zoonoses & Tropical Veterinary Medicine, Ross University School of Veterinary Medicine, Saint Kitts

^4^Department of Veterinary Medicine, College of Agriculture & Veterinary Medicine, United Arab Emirates

*corresponding authors: JLN Barratt

ORCID ID of JLN Barratt: 0000-0001-8711-2408

**Email (J. L. N. Barratt):** [jbarratt@cdc.gov](mailto:jbarratt@cdc.gov)

# Table of contents

[Genetic characterization of *Strongyloides fuelleborni* infecting free-roaming African vervets (*Chlorocebus aethiops sabaeus*) on the Caribbean island of St. Kitts i](#_Toc124233710)

[Table of contents ii](#_Toc124233711)

[Appendix A. Establishing Minimum data requirements and cox1 haplotype definitions 1](#_Toc124233712)

[Haplotype definitions and maximum imputation limits 1](#_Toc124233713)

[Figure S1. Graphical depiction of cox1 amplicons generated in various studies relative to the 15-mer segments used in the present study for distance computation 4](#_Toc124233714)

[Minimum data requirements 4](#_Toc124233715)

[Appending A References 5](#_Toc124233716)

[Appendix B. Fasta sequences of each HVR-I haplotype 7](#_Toc124233717)

[Appendix C. Fasta sequences of each HVR-IV haplotype 9](#_Toc124233718)

[Appendix D. Fasta sequences of segmented haplotypes 11](#_Toc124233719)

#

# Appendix A. Establishing Minimum data requirements and cox1 haplotype definitions

## Haplotype definitions and maximum imputation limits

Haplotype definitions for HVR-I and HVR-IV are identical to those from Barratt and Sapp (2020) (Figure 1, Appendices B, C, and D). For cox1, we defined haplotypes differently to what was originally described by Barratt and Sapp (2020). As done previously (Barratt and Sapp, 2020, Jacobson et al., 2022), the cox1 locus was divided into segments and haplotypes were named/defined separately (i.e., as a distinct marker) for each segment. However, in this study the cox1 locus was divided into segments of 15 bases, which is ~10 bases shorter than what was used previously (Barratt and Sapp, 2020); this was done to increase granularity in the tree structure (Jacobson et al., 2022). Additionally, Barratt and Sapp (2020) only considered a 217 bp region of cox1 during distance computation. The cox1 sequences included in this study covered different parts of cox1: some investigators sequenced ~700 to 800 base pairs (bp) (Frias et al., 2018, Janwan et al., 2020), some ~550 bp (Jaleta et al., 2017, Zhou et al., 2019), while others – including the present study -- sequenced 217 bp (Barratt et al., 2019, Beknazarova et al., 2019). Here, we attempted to take greater advantage of the imputation abilities of Barratt’s heuristic by increasing the portion of cox1 included during distance computation. However, prior to distance computation, maximum imputation limits (i.e., how much of cox1 we would allow Barratt’s heuristic to attempt to impute) would be needed to be established to ensure a robust phylogenetic reconstruction. Note that considering the complete cox1 gene during distance computation would require Barratt’s heuristic to impute values for up to 38 of 52 (~73%) cox1 segments for isolates sequenced at the shortest 217 bp fragment, such that a potentially tenuous tree structure was expected.

To establish maximum imputation limits, a complete cox1 gene was extracted from the mitochondrial genome of *S. stercoralis* strain PV001 (GB: NC_028624.1), which has a coding sequence starting at base 5381 and ending at base 6922. Cox1 sequences from all reference types were aligned to this region of NC_028624.1 using MUSCLE (MUltiple Sequence Comparison by Log- Expectation) (Edgar, 2004). These sequences aligned to the PV001 reference at various locations between positions 5623 and 6404 relative to NC_028624.1 (Figure S1 – below). Therefore, we divided this region (positions 5624 to 6403) into 52 k-mers of 15 bases each. For the purposes of HDS construction, these 52 cox1 segments were assigned a unique identifier (i.e., A1, A2, A3, B2, B2, B3, etcetera, through to segment R1) and all unique 15-mer haplotypes aligning to each segment were extracted from the larger alignment and assigned a sequential haplotype number (e.g., A1_hap_1, A1_hap_2, etc.). The 217 bp fragment sequenced here includes the 14 segments starting at J3 and ending at O1 (210 bp), as depicted in Figure S1.

We next computed a distance matrix using Barratt’s heuristic (methods provided in main manuscript text). A HDS was generated to list all known haplotypes from all *Strongyloides* isolates analyzed here (from vervets and references types), including data from both HVRs and cox1 segments A1 through R1 identified from the MUSCLE alignment (See HDS, File S1, Tab D). A pairwise distance matrix was computed from this HDS (File S1, Tab E), and the matrix was hierarchically clustered (methods provided in main manuscript text) to produce a tree. We assessed the robustness of this ‘52 segment’ tree structure against the following criteria which are based on prescient knowledge of the relationships between the *S. stercoralis* and *S. fuelleborni* isolates from our reference population:

a) *S. stercoralis* type B must be monophyletic and cluster separately to isolates of *S. stercoralis* type A (Jaleta et al., 2017).

b) The *Strongyloides* sp. identified in lorises by Frias et al. (2018) must cluster as a sister group to *S. stercoralis* (Frias et al., 2018).

c) The *S. stercoralis*/*Strongyloides* sp. Loris clade/s must be monophyletic and distinct from all *S. fuelleborni* types (Ko et al., 2020).

d) African *S. fuelleborni* should be monophyletic and distinct from Asian types (Ko et al., 2022).

e) *S. fuelleborni* from Japanese macaques should be monophyletic (Ko et al., 2022).

f) *S.* *fuelleborni* from West/Central Africa should be distinct from Tanzanian/East African types (Ko et al., 2022).

After establishing these criteria, 15-mer segments were iteratively excluded from either end of the 52-segment cox1 reference region (starting with segment A1) by removing all haplotypes detected at a given segment from the HDS. With each excluded segment, a new genetic distance matrix was computed from the updated HDS to produce a tree. Each new tree was evaluated against the criteria listed above, until a tree structure was obtained where all criteria were satisfied. At this point, the maximum imputation limit was reached and downstream analyses only included the largest number of cox1 segments resulting that tree structure meeting the above criteria. This process served to calibrate this analysis against relationships already well-established for published reference isolates. In turn, it ensured that our *S. fuelleborni* types from St Kitts vervets would be placed accurately within our final phylogenetic tree. Note that segments excluded via this calibration process did not contribute to the minimum requirement of 14 segments (as described below), so isolates with fewer than 14 cox1segments remaining after these exclusions were excluded from our final analysis.


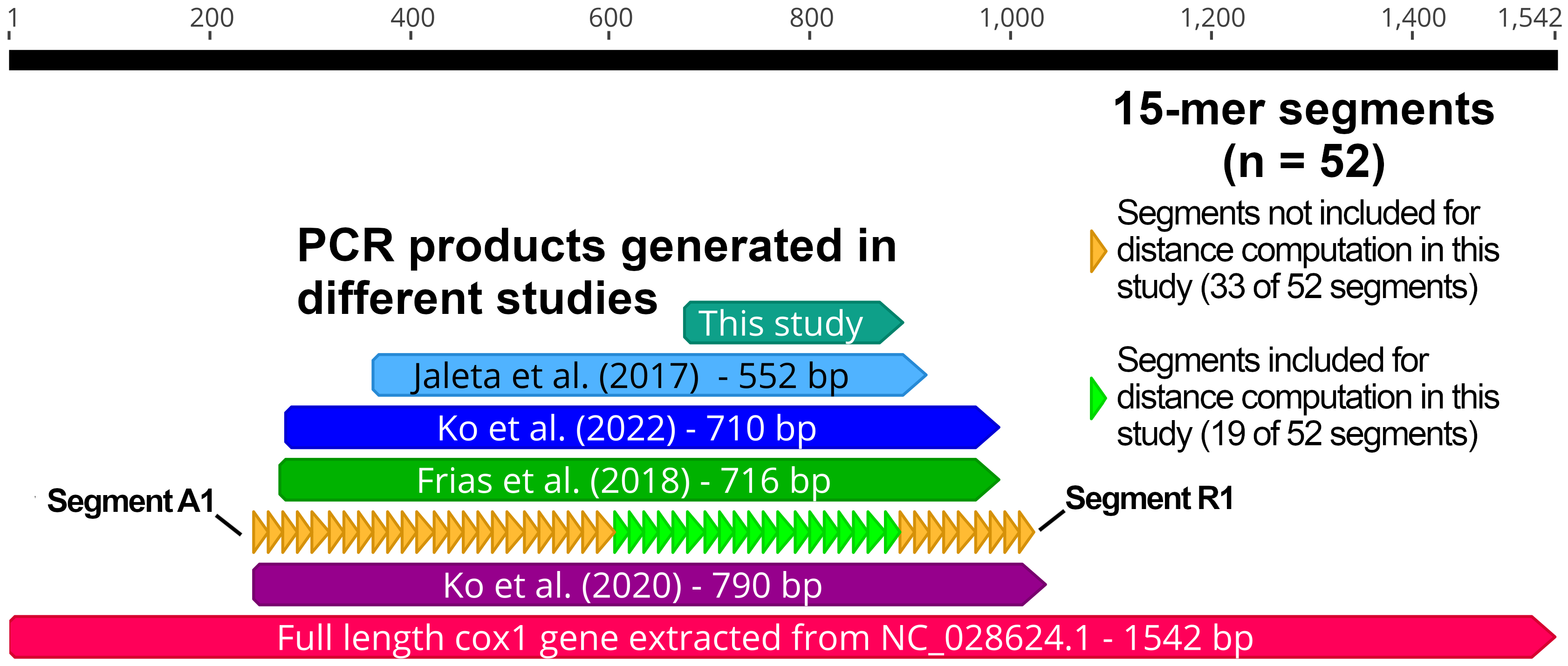


### Figure S1. Graphical depiction of cox1 amplicons generated in various studies relative to the 15-mer segments used in the present study for distance computation

Difference regions of the *Strongyloides* cox1 gene were sequenced in various independent studies. In the present study, a cox1 region of 217 base pairs was sequenced. Ko et al. (2020) published amplicon sequences of cox1 that spanned the largest portion (790 base pairs – bp) of the full-length cox1 gene. Therefore, we divided this portion of the cox1 locus into 52 segments of 15 bases in length (segments A1, A2, A3, through to R1) and defined novel haplotypes at each of these segments using the methods described above. Following the iterative calibration process described above, we observed that considering segments I1 through O1 for distance computation (i.e., 19 segments, or 285 bp – green triangles above) resulted in a robust phylogeny that met the above criteria (see Results, main manuscript text). Consequently, only these segments of cox1 (green) were considered. This would require Barratt’s heuristic to impute distances for 5 segments beyond the 14 segments sequenced in the present study.

## Minimum data requirements

For a genotype to be included in this analysis, a cox1 sequence was an absolute requirement. Isolates sequenced at only the HVRs were excluded from our reference dataset because cox1 has a higher entropy than the HVRs and therefore contains the most genotypic information (Barratt and Sapp, 2020). Cox1 is also the most widely sequenced marker across numerous studies (Barratt et al., 2019, Beknazarova et al., 2019, Barratt and Sapp, 2020, Janwan et al., 2020, Ko et al., 2022, Jaleta et al., 2017). As described above, the cox1 locus was divided into 15-mer segments. If an isolate possessed at least 14 cox1 segments, it was retained for distance computation. If an isolate possessed 13 cox1 segments, it would only be retained if it also had data for segments B and C of HVR-IV (Figure 1) (Barratt and Sapp, 2020). If an isolate’s cox1 sequence had fewer than 13 segments, it was excluded.

Reference *Strongyloides* sp. genotypes had been sequenced at only HVR-IV, only HVR-I, both HVRs, or neither. Isolates possessing at least 14 cox1 segments were retained for distance computation regardless of whether they possessed accompanying HVR data or not, noting that any available HVR data would still be included in the HDS if available.

## Appending A References

BARRATT, J. L. N., LANE, M., TALUNDZIC, E., RICHINS, T., ROBERTSON, G., FORMENTI, F., PRITT, B., VEROCAI, G., NASCIMENTO DE SOUZA, J., MATO SOARES, N., TRAUB, R., BUONFRATE, D. & BRADBURY, R. S. 2019. A global genotyping survey of *Strongyloides stercoralis* and *Strongyloides fuelleborni* using deep amplicon sequencing. *PLoS Negl Trop Dis,* 13**,** e0007609.

BARRATT, J. L. N. & SAPP, S. G. H. 2020. Machine learning-based analyses support the existence of species complexes for *Strongyloides fuelleborni* and *Strongyloides stercoralis*. *Parasitology,* 147**,** 1184-1195.

BEKNAZAROVA, M., BARRATT, J., BRADBURY, R., LANE, M., WHILEY, H. & ROSS, K. 2019. Detection of classic and cryptic *Strongyloides* genotypes by deep amplicon sequencing: A preliminary survey of dog and human specimens collected from remote Australian communities. *PLoS neglected tropical diseases,* Under review.

EDGAR, R. C. 2004. MUSCLE: multiple sequence alignment with high accuracy and high throughput. *Nucleic Acids Res,* 32**,** 1792-7.

FRIAS, L., STARK, D. J., LYNN, M. S., NATHAN, S. K., GOOSSENS, B., OKAMOTO, M. & MACINTOSH, A. J. J. 2018. Lurking in the dark: Cryptic *Strongyloides* in a Bornean slow loris. *Int J Parasitol Parasites Wildl,* 7**,** 141-146.

JACOBSON, D., ZHENG, Y., PLUCINSKI, M. M., QVARNSTROM, Y. & BARRATT, J. L. N. 2022. Evaluation of various distance computation methods for construction of haplotype-based phylogenies from large MLST datasets. *Mol Phylogenet Evol,* 177**,** 107608.

JALETA, T. G., ZHOU, S., BEMM, F. M., SCHAR, F., KHIEU, V., MUTH, S., ODERMATT, P., LOK, J. B. & STREIT, A. 2017. Different but overlapping populations of *Strongyloides stercoralis* in dogs and humans-Dogs as a possible source for zoonotic strongyloidiasis. *PLoS Negl Trop Dis,* 11**,** e0005752.

JANWAN, P., RODPAI, R., INTAPAN, P. M., SANPOOL, O., TOURTIP, S., MALEEWONG, W. & THANCHOMNANG, T. 2020. Possible transmission of *Strongyloides fuelleborni* between working Southern pig-tailed macaques (Macaca nemestrina) and their owners in Southern Thailand: Molecular identification and diversity. *Infect Genet Evol,* 85**,** 104516.

KO, P. P., HARAGUCHI, M., HARA, T., HIEU, D. D., ITO, A., TANAKA, R., TANAKA, M., SUZUMURA, T., UEDA, M., YOSHIDA, A., MARUYAMA, H. & NAGAYASU, E. 2022. Population genetics study of *Strongyloides fuelleborni* and phylogenetic considerations on primate-infecting species of *Strongyloides* based on their mitochondrial genome sequences. *Parasitol Int,* 92**,** 102663.

KO, P. P., SUZUKI, K., CANALES-RAMOS, M., AUNG, M., HTIKE, W. W., YOSHIDA, A., MONTES, M., MORISHITA, K., GOTUZZO, E., MARUYAMA, H. & NAGAYASU, E. 2020. Phylogenetic relationships of *Strongyloides* species in carnivore hosts. *Parasitol Int,* 78**,** 102151.

ZHOU, S., FU, X., PEI, P., KUCKA, M., LIU, J., TANG, L., ZHAN, T., HE, S., CHAN, Y. F., RODELSPERGER, C., LIU, D. & STREIT, A. 2019. Characterization of a non-sexual population of *Strongyloides stercoralis* with hybrid 18S rDNA haplotypes in Guangxi, Southern China. *PLoS Negl Trop Dis,* 13**,** e0007396.

# Appendix B. Fasta sequences of each HVR-I haplotype

>Haplotype_I

AATATTTTAGTTGGATAACTGAGGTAATTCTTGAGCTAATACACGCTATTTATACCACATTAGTGGTGCGTTTATTTGATTAAACCATTTTATATTGGTTGACTCAAAATATCCTCGCTGATTTTGTTACTAAAACATACCGTATGTGTATCTGGTTTATCAACTTTCGATGGTAGGGTATTGGCCTACCATGGTTGTGACGGATAACGGAGAATTAGGGTTCGACTCCGGAGAGGGAGCCTGAGAAACGGCTACCACATCCAAGGAAGGCAGCAGGCGCGAAAATTACCCAATTTTAGTTAAAAGAGGTAGTGACGAAAAATGACAACCAAATATTATTATTAATATTTGGATTGAAAATCTTCAAGTTTAAATAACTTGTTGGTAAAGGAAAGGGCAAGTCTGGTGCCAGCAGCCGCGGTAATACCAGCTTT

>Haplotype_II

AATATTTTAGTTGGATAACTGAGGTAATTCTTGAGCTAATACACGCTATTTATACCACATTAGTGGTGCGTTTATTTGATTAAACCATTTTTATATTGGTTGACTCAAAATATCCTCGCTGATTTTGTTACTAAAACATACCGTATGTGTATCTGGTTTATCAACTTTCGATGGTAGGGTATTGGCCTACCATGGTTGTGACGGATAACGGAGAATTAGGGTTCGACTCCGGAGAGGGAGCCTGAGAAACGGCTACCACATCCAAGGAAGGCAGCAGGCGCGAAAATTACCCAATTTTAGTTAAAAGAGGTAGTGACGAAAAATGACAACCAAATATTATTATTAATATTTGGATTGAAAATCTTCAAGTTTAAATAACTTGTTGGTAAAGGAAAGGGCAAGTCTGGTGCCAGCAGCCGCGGTAATACCAGCTTT

>Haplotype_III

AATATTTTAGTTGGATAACTGAGGTAATTCTTGAGCTAATACACGCTATTTATACCACATTAGTGGTGCGTTTATTTGATTAAACCATTTTTATATTGGTTGACTCAAAATATCCTCGCTGATTTTGTTACTAAAACATACCGTATGTGTATCTGGTTTATCAACTTTCGATGGTAGGGTATTGGCCTACCATGGTTGTGACGGATAACGGAGAATTAGGGTTCGACTCCGGAGAGGGAGCCTGAGAAACGGCTACCACATCCAAGGAAGGCAGCAGGCGCGAAAATTACCCAATTTTAGTTAAAAGAGGTAGTGACGAAAAATGACAACCAAATATTATTATTAATATTTGGATTGAAAATCTTCAAGTATAAATAACTTGTTGGTAAAGGAAAGGGCAAGTCTGGTGCCAGCAGCCGCGGTAATACCAGCTTT

>Haplotype_IV

AATATTTTAGTTGGATAACTGAGGTAATTCTTGAGCTAATACACGCTATTTATACCACATTAGTGGTGCGTTTATTTGATTAAACCATTTATACTGGTTGACTCAAAATATCCTCGCTGATTTTGTTACTAAAACATACCGTATGTGTATCTGGTTTATCAACTTTCGATGGTAGGGTATTGGCCTACCATGGTTGTGACGGATAACGGAGAATTAGGGTTCGACTCCGGAGAGGGAGCCTGAGAAACGGCTACCACATCCAAGGAAGGCAGCAGGCGCGAAAATTACCCAATTTTAGTTAAAAGAGGTAGTGACGAAAAATGACAACCAAATATTATTATTAATATTTGGATTGAAAATCTTCAAGTTTAAATAACTTGTTGGTAAAGGAAAGGGCAAGTCTGGTGCCAGCAGCCGCGGTAATACCAGCTTT

>Haplotype_V

AATATTTTAGTTGGATAACTGAGGTAATTCTTGAGCTAATACACGCTATTTATACCACATTAGTGGTGCGTTTATTTGATTAAACCATTTTATACTGGTTGACTCAAAATATCCTCGCTGATTTTGTTACTAAAACATACCGTATGTGTATCTGGTTTATCAACTTTCGATGGTAGGGTATTGGCCTACCATGGTTGTGACGGATAACGGAGAATTAGGGTTCGACTCCGGAGAGGGAGCCTGAGAAACGGCTACCACATCCAAGGAAGGCAGCAGGCGCGAAAATTACCCAATTTTAGTTAAAAGAGGTAGTGACGAAAAATGACAACCAAATATTATTATTAATATTTGGATTGAAAATCTTCAAGTTTAAATAACTTGTTGGTAAAGGAAAGGGCAAGTCTGGTGCCAGCAGCCGCGGTAATACCAGCTTT

>Haplotype_VI

AATATTTTAGTTGGATAACTGAGGTAATTCTTGAGCTAATACACGCTATTTATACCACATTAGTGGTGCGTTTATTTGATTAAACCATTTTATATTGGTTGACTCAAAATATCCTCGCTGATTTTGTTACTAAAACATACCGTATGTGTATCTGGTTTATCAACTTTCGATGGTAGGGTATTGGCCTACCATGGTTGTGACGGATAACGGAGAATTAGGGTTCGACTCCGGAGAGGGAGCCTGAGAAACGGCTACCACATCCAAGGAAGGCAGCAGGCGCGAAAATTACCCAATTTTAGTTAAAAGAGGTAGTGACGAAAAATGACAACCAAATATTATTATTAATATTTGGATTGAAAATCTTCAAGTATAAATAACTTGTTGGTAAAGGAAAGGGCAAGTCTGGTGCCAGCAGCCGCGGTAATACCAGCTTT

>Haplotype_VII

AATATTTTAGTTGGATAACTGAGGTAATTCTTGAGCTAATACACGCTATTTATACCACATTAGTGGTGCGTTTATTTGATTAAACCATTTATATTGGTTGACTCAAAATATCCTCGCTGATTTTGTTACTAAAACATACCGTATGTGTATCTGGTTTATCAACTTTCGATGGTAGGGTATTGGCCTACCATGGTTGTGACGGATAACGGAGAATTAGGGTTCGACTCCGGAGAGGGAGCCTGAGAAACGGCTACCACATCCAAGGAAGGCAGCAGGCGCGAAAATTACCCAATTTTAGTTAAAAGAGGTAGTGACGAAAAATGACAACCAAATATTATTATTAATATTTGGATTGAAAATCTTCAAGTTTAAATAACTTGTTGGTAAAGGAAAGGGCAAGTCTGGTGCCAGCAGCCGCGGTAATACCAGCTTT

>Haplotype_VIII

AATATTTTAGTTGGATAACTGAGGTAATTCTTGAGCTAATACACGCTGTTAATACCACATTAGTGGTGCGTTTATTTGATTAAACCATTATATTGGTTGACTCAAAATATCCTCGCTGATTTTGTTACTAAAACATACCGTATGTGTATCTGGTTTATCAACTTTCGATGGTAGGGTATTGGCCTACCATGGTTGTGACGGATAACGGAGAATTAGGGTTCGACTCCGGAGAGGGAGCCTGAGAAACGGCTACCACATCCAAGGAAGGCAGCAGGCGCGAAAATTACCCAATTTTAGTTAAAAGAGGTAGTGACGAAAAATGACAACCAAATATTATTATTAATATTTGGATTGAAAATCTTCAAGTTTAAATAACTTGTTGGTAAAGGAAAGGGCAAGTCTGGTGCCAGCAGCCGCGGTAATACCAGCTTT

>Haplotype_IX

AATATTTTAGTTGGATAACTGAGGTAATTCTTGAGCTAATACACGCTACCTATACCACATTAGTGGTGCGTTTATTTGATTAAACCATTATTTTGGTTGACTCAAAATATCCTCGCTGATTTTGTTACTAAAACATACCGTATGTGTATCTGGTTTATCAACTTTCGATGGTAGGGTATTGGCCTACCATGGTTGTGACGGATAACGGAGAATTAGGGTTCGACTCCGGAGAGGGAGCCTGAGAAACGGCTACCACATCCAAGGAAGGCAGCAGGCGCGAAAATTACCCAATTTTAGTTTAAAGAGGTAGTGACGAAAAATGACAACCAAATATTATTATTAATATTTGGATTGAAAATCTTCAAGTTTAAATAACTTGTTGGTAAAGGAAAGGGCAAGTCTGGTGCCAGCAGCCGCGGTAATACCAGCTTT

>Haplotype_X

ACTATTTTAGTTGGATAACTGAGGTAATTCTTGAGCTAATACACGCTATTTATACCACATTAGTGGTGCGTTTATTTGATTAAACCATTATTTTGGTTGACTCAAAATATCCTCGCTGATTTTGTTACAAAAACATACCGTATGTGTATCTGGTTTATCAACTTTCGATGGTAGGGTATTGGCCTACCATGGTTGTGACGGATAACGGAGAATTAGGGTTCGACTCCGGAGAGGGAGCCTGAGAAACGGCTACCACATCCAAGGAAGGCAGCAGGCGCGAAAATTACCCAATTTTAGTTTAAAGAGGTAGTGACGAAAAATGACAACCAAATATTATTATTAATATTTGGATTGAAAATCTTCAAGTTTAAATAACTTGTTGGTAAAGGAAAGGGCAAGTCTGGTGCCAGCAGCCGCGGTAATACCAGCTTT

>Haplotype_XI

AATATTTTAGTTGGATAACTGAGGTAATTCTTGAGCTAATACACGCTATTTATACCACATTAGTGGTGCGTTTATTTGATTAAACCATTTTTATATATTGGTTGACTCAAAATATCCTCGCTGATTTTGTTACTAAAACATACCGTATGTGTATCTGGTTTATCAACTTTCGATGGTAGGGTATTGGCCTACCATGGTTGTGACGGATAACGGAGAATTAGGGTTCGACTCCGGAGAGGGAGCCTGAGAAACGGCTACCACATCCAAGGAAGGCAGCAGGCGCGAAAATTACCCAATTTTAGTTAAAAGAGGTAGTGACGAAAAATGACAACCAAATATTATTATTAATATTTGGATTGAAAATCTTCAAGTTTAAATAACTTGTTGGTAAAGGAAAGGGCAAGTCTGGTGCCAGCAGCCGCGGTAATACCAGCTTT

>Haplotype_XII

AATATTTTAGTTGGATAACTGAGGTAATTCTTGAGCTAATACACGCTATTTATACCACATTAGTGGTGCGTTTATTTGATTAAACCATTATAACGGTTGACTCAAAATATCCTTGCTGATTTTGTTACTAAAACATACCGTATGTGTATCTGGTTTATCAACTTTCGATGGTAGGGTATTGGCCTACCATGGTTGTGACGGATAACGGAGAATTAGGGTTCGACTCCGGAGAGGGAGCCTGAGAAACGGCTACCACATCCAAGGAAGGCAGCAGGCGCGAAAATTACCCAATTTTAGTTCAAAGAGGTAGTGACGAAAAATGACAACCAAATATTATTATTAATATTTGGATTGAAAATCTTCAAGTTTAAATAACTTGTTGGTAAAGGAAAGGGCAAGTCTGGTGCCAGCAGCCGCGGTAATACCAGCTTT

>Haplotype_XIII

GTTGGATAACTGACGTAATTCTTGAGCTAATACACGCTATTTATACCACATTAGTGGTGCGTTTATTTGATTAAACCATTATAACGGTTGACTCAAAATATCCTTGCTGATTTTGTTACTAAAACATACCGTATGTGTATCTGGTTTATCAACTTTCGATGGTAGGGTATTGGCCTACCATGGTTGTGACGGATAACGGAGAATTAGGGTTCGACTCCGGAGAGGGAGCCTGAGAAACGGCTACCACATCCAAGGAAGGCAGCAGGCGCGAAAATTACCCAATTTTAGTTCAAAGAGGTAGTGACGAAAAATGACAACCAAATATTATTATTAATATTTGGATTGAAAATCTTCAAGTTTAAATAACTTGTTGGTAAAGGTTTGGGCAAGTCTGGTGCCAGCAGCCGCGGTAATACCAGCTTT

>Haplotype_XIV

AATATTTTAGTTGGATAACTGAGGTAATTCTTGAGCTAATACACGCTATTTATACCACATTAGTGGTGCGTTTATTTGATTAAACCATTTTAACGGTTGACTCAAAATATCCTTGCTGATTTTGTTACTAAAACATACCGTATGTGTATCTGGTTTATCAACTTTCGATGGTAGGGTATTGGCCTACCATGGTTGTGACGGATAACGGAGAATTAGGGTTCGACTCCGGAGAGGGAGCCTGAGAAACGGCTACCACATCCAAGGAAGGCAGCAGGCGCGAAAATTACCCAATTTTAGTTCAAAGAGGTAGTGACGAAAAATGACAACCAAATATTATTATTAATATTTGGATTGAAAATCTTCAAGTTTAAATAACTTGTTGGTAAAGGAAAGGGCAAGTCTGGTGCCAGCAGCCGCGGTAATACCAGCTTT

>Haplotype_XV

AATATTTTAGTTGGATAACTGAGGTAATTCTTGAGCTAATACACGCTATTTATACCACATTAGTGGTGCGTTTATTTGATTAAACCATTTTTATATTGGTTGACTCAAAATATCCTCGCTGATTTTGTTACTAAAACATACCGTATGTGTATCTGGTTTATCAACTTTCGATGGTAGGGTATTGGCCTACCATGGTTGTGACGGATAACGGAGAATTAGGGTTCGACTCCGGAGAGGGAGCCTGAGAAATGGCTACCACATCCAAGGAAGGCAGCAGGCGCGAAAATTACCCAATTTTAGTTAAAAGAGGTAGTGACGAAAAATGACAACCAAATATTATTATTAATATTTGGATTGAAAATCTTCAAGTATAAATAACTTGTTGGTAAAGGAAAGGGCAAGTCTGGTGCCAGCAGCCGCGGTAATACCAGCTTT

>Haplotype_XVI

AATATTTTAGTTGGATAACTGAGGTAATTCTTGAGCTAATACACGCTATTTATACCACATTAGTGGTGCGTTTATTTGATTAAACCATTATAACGGTTGACTCAAAATATCCTTGCTGATTTTGTTACTAAAACATACCGTATGTGTATCTGGTTTATCAACTTTCGATGGTAGGGTATTGGCCTACCATGGTTGTGACGGATAACGGAGAATTAGGGTTCGACTCCGGAGAGGGAGCCTGAGAAACGGCTACCACATCCAAGGAAGGCAGCAGGCGCGAAAATTACCCAATTTTAGTTCAAAGAGGTAGTGACGAAAAATGACAACCAAATATTATTATATTAATATTTGGATTGAAAATCTTCAAGTTTAAATAACTTGTTGGTAAAGGAAAGGGCAAGTCTGGTGCCAGCAGCCGCGGTAATACCAGCTTT

>Haplotype_XVII

AATATTTTAGTTGGATAACTGAGGTAATTCTTGAGCTAATACACGCTATTTATACCACATTAGTGGTGCGTTTATTTGATTAAACCATTATATAACGGTTGACTCAAAATATCCTTGCTGATTTTGTTACTAAAACATACCGTATGTGTATCTGGTTTATCAACTTTCGATGGTAGGGTATTGGCCTACCATGGTTGTGACGGATAACGGAGAATTAGGGTTCGACTCCGGAGAGGGAGCCTGAGAAACGGCTACCACATCCAAGGAAGGCAGCAGGCGCGAAAATTACCCAATTTTAGTTCAAAGAGGTAGTGACGAAAAATGACAACCAAATATTATTATTAATATTTGGATTGAAAATCTTCAAGTTTAAATAACTTGTTGGTAAAGGAAAGGGCAAGTCTGGTGCCAGCAGCCGCGGTAATACCAGCTTT

# Appendix C. Fasta sequences of each HVR-IV haplotype

>Haplotype_A

ATTGACAGATTGATAGCTCTTTCATGATTTAGTGGTTGGTGGTGCATGGCCGTTCTTAGTTCGTGGATATGATTTGTCTGGTTGATTCCGATAACGAGCGAGACTTTTATGTTATATTAAATATTATTATTTTGTTTATTTTAATATAAATAATTAATATTTTAATAACAGATTAATAGTGTTTAACTATTTGAGAGAGAGCGATAACAGGTCTGTGATGCCCTTAGATGTCCGGGGCTGCACGCGCGCTACAAT

>Haplotype_B

ATTGACAGATTGATAGCTCTTTCATGATTTAGTGGTTGGTGGTGCATGGCCGTTCTTAGTTCGTGGATATGATTTGTCTGGTTGATTCCGATAACGAGCGAGACTTTTATGTTATATTAAATATTATTATTTGTTTATTTTTTATATAAATAATTAATATTTTAATAACAGATTAATAGTGTTTAACTATTTGAGAGAGAGCGATAACAGGTCTGTGATGCCCTTAGATGTCCGGGGCTGCACGCGCGCTACAAT

>Haplotype_C

ATTGACAGATTGATAGCTCTTTCATGATTTAGTGGTTGGTGGTGCATGGCCGTTCTTAGTTCGTGGATATGATTTGTCTGGTTGATTCCGATAACGAGCGAGACTTTTATGTTATATTAAATATAATTATTTTGTTTATTTTAATATAAATAATTAATATTTTAATAACAGATTAATAGTGTTTAACTATTTGAGAGAGAGCGATAACAGGTCTGTGATGCCCTTAGATGTCCGGGGCTGCACGCGCGCTACAAT

>Haplotype_D

ATTGACAGATTGATAGCTCTTTCATGATTTAGTGGTTGGTGGTGCATGGCCGTTCTTAGTTCGTGGATATGATTTGTCTGGTTGATTCCGATAACGAGCGAGACTTTTATGTTATATTAAATATTATTATTTGTTTATTTTATATAAATAATTAATATTTTAATAACAGATTAATAGTGTTTAACTATTTGAGAGAGAGCGATAACAGGTCTGTGATGCCCTTAGATGTCCGGGGCTGCACGCGCGCTACAAT

>Haplotype_E

ATTGACAGATTGATAGCTCTTTCATGATTTAGTGGTTGGTGGTGCATGGCCGTTCTTAGTTCGTGGATATGATTTGTCTGGTTGATTCCGATAACGAGCGAGACTTTTATGTTATATTAAATATTATTATTTGTTTATTTTAATATAAATAATTAATATTTTAATAACAGATTAATAGTGTTTAACTATTTGAGAGAGAGCGATAACAGGTCTGTGATGCCCTTAGATGTCCGGGGCTGCACGCGCGCTACAAT

>Haplotype_F

ATTGACAGATTGATAGCTCTTTCATGATTTAGTGGTTGGTGGTGCATGGCCGTTCTTAGTTCGTGGATATGATTTGTCTGGTTGATTCCGATAACGAGCGAGACTTTTATGTTATATTAAATATTATTATTTTATTATTTTATATAAATAATTAATATTTTAATAACAGATTAATAGTGTTTAACTATTTGAGAGAGAGCGATAACAGGTCTGTGATGCCCTTAGATGTCCGGGGCTGCACGCGCGCTACAAT

>Haplotype_G

ATTGACAGATTGATAGCTCTTTCATGATCTAGTGGTTGGTGGTGCATGGCCGTTCTTAGTTCGTGGATATGATTTGTCTGGTTGATTCCGATAACGAGCGAGACTTTTATGTTATATTAAATATTATTATTTTGTTTATTTTAATATAAATAATTAATATTTTAATAACAGATTAATAGTGTTTAACTATTTGAGAGAGAGCGATAACGGGTCTGTGATGCCCTTAGATGTCCGGGGCTGCACGCGCGCTACAAT

>Haplotype_H

ATTGACAGATTGATAGCTCTTTCATGATTTAGTGGTTGGTGGTGCATGGCCGTTCTTAGTTCGTGGATATGATTTGTCTGGTTGATTCCGATAACGAGCGAGACTTTTATGTTATATTAAATATTATTATTTTATTATATAAATAATAATTATTTTAATAACAGATTAATAGTGTTTAACTATTTGAGAGAGAGCAATAACAGGTCTGTGATGCCCTTAGATGTCCGGGGCTGCACGCGCGCTACAAT

>Haplotype_I

ATTGACAGATTGATAGCTCTTTCATGATTTAGTGGTTGGTGGTGCATGGCCGTTCTTAGTTCGTGGATATGATTTGTCTGGTTGATTCCGATAACGAGCGAGACTTTTATGTTATATTAAATATTATTATTTTTTATATAAATAATAATTATTTTAATAACAGATTAATAGTGTTTAACTATTTGAGAGAGAGCAATAACAGGTCTGTGATGCCCTTAGATGTCCGGGGCTGCACGCGCGCTACAAT

>Haplotype_J

ATTGACAGATTGATAGCTCTTTCATGATTTAGTGGTTGGTGGTGCATGGCCGTTCTTAGTTCGTGGATATGATTTGTCTGGTTGATTCCGATAACGAGCGAGACTTTTATGTTATATTAAATATTATTATTTTGTTTATTTTAATATAAATAATTAATATTTTAATAACAGATTAATAGTGTTTAACTATTTGAGAGAGAGCGATAACAGGTATGTGATGCCCTTAGATGTCCGGGGCTGCACGCGCGCTACAAT

>Haplotype_K

ATTGACAGATTGATAGCTCTTTCATGATTTAGTGGTTGGTGGTGCATGGCCGTTCTTAGTTCGTGGATATGATTTGTCTGGTTGATTCCGATAACGAGCGAGACTTTTATGTTATATTAAATAATATTATTTATAAATTTTATATTTTATATAATATTTTATTTTAATAACAGATTAATAGTGTTTAACTATTTGAGAGAGAGCAATAACAGGTCTGTGATGCCCTTAGATGTCCGGGGCTGCACGCGCGCTACAAT

>Haplotype_L

ATTGACAGATTGATAGCTCTTTCATGATTTAGTGGTTGGTGGTGCATGGCCGTTCTTAGTTCGTGGATATGATTTGTCTGGTTGATTCCGATAACGAGCGAGACTTTTATGTTATATTAAATAATATTATTGTTAAATTTTATTTTTATATAATATTTTATTTTAATAACAGATTAATAGTGTTTAACTATTTGAGAGAGAGCAATAACAGGTCTGTGATGCCCTTAGATGTCCGGGGCTGCACGCGCGCTACAAT

>Haplotype_M

ATTGACAGATTGATAGCTCTTTCATGATTTAGTGGTTGGTGGTGCATGGCCGTTCTTAGTTCGTGGATATGATTTGTCTGGTTGATTCCGATAACGAGCGAGACTTTTATGTTATATTAAATAATATTATTTATAAATTTTATAGTTTTTATATAATATTTTATTTTAATAACAGATTAATAGTGTTTAACTATTTGAGAGAGAGCAATAACAGGTCTGTGATGCCCTTAGATGTCCGGGGCTGCACGCGCGCTACAAT

>Haplotype_N

ATTGACAGATTGATAGCTCTTTCATGATTTAGTGGTTGGTGGTGCATGGCCGTTCTTAGTTCGTGGATATGATTTGTCTGGTTGATTCCGATAACGAGCGAGACTTTTATGTTATATTAAATAATATTATTTATAAATTTTATATTTTATATAATATTTTATTTTAATAACAGATTAATAGTGCGTAACTA

>Haplotype_O

ATTGACAGATTGATAGCTCTTTCATGATTTAGTGGTTGGTGGTGCATGGCCGTTCTTAGTTCGTGGATATGATTTGTCTGGTTGATTCCGATAACGAGCGAGACTTTTATGTTATATTAAATAATATTATTGTTAAATTTTATGGTTTTATATAATATTTTATTTTAATAACAGATTAATAGTGTTTAACTATTTGAGAGAGAGCAATAACAGGTCTGTGATGCCCTTAGATGTCCGGGGCTGCACGCGCGCTACAAT

>Haplotype_P

ATTGACAGATTGATAGCTCTTTCATGATTTAGTGGTTGGTGGTGCATGGCCGTTCTTAGTTCGTGGATATGATTTGTCTGGTTGATTCCGATAACGAGCGAGACTTTTATGTTATATTAAATAATATTATTATTAAATTTTATTTTATATAATATTTTATTTTAATAACAGATTAATAGTGTTTAACTATTTGAGAGAGAGCAATAACAGGTCTGTGATGCCCTTAGATGTCCGGGGCTGCACGCGCGCTACAAT

>Haplotype_Q

ATTGACAGAGTGATAGCTCTTTCATGATTTAGTGGTTGGTGGTGCATGGCCGTTCTTAGTTCGTGGATATGATTTGTCTGGTTGATTCCGATAACGAGCGAGACTTTTATGTTATATTAAATAATATTATTTATAAATTTTATATATTATATAATATTTTATTTTAATAACAGATTAATAGTGTTTAACTATTTGAGAGAGAGCAATAACAGGTCTGTGA

>Haplotype_R

ATTGACAGATTGATAGCTCTTTCATGATTTAGTGGTTGGTGGTGCATGGCCGTTCTTAGTTCGTGGATATGATTTGTCTGGTTGATTCCGATAACGAGCGAGACTTTTATGTTATATTAAATAATATTATTATTAAATTTTATGGTTTTATATAATATTTTATTTTAATAACAGATTAATAGTGTTTAACTATTTGAGAGAGAGCAATAACAGGTCTGTGATGCCCTTAGATGTCCGGGGCTGCACGCGCGCTACAAT

>Haplotype_S

ATTGACAGATTGATAGCTCTTTCATGATTTAGTGGTTGGTGGTGCATGGCCGTTCTTAGTTCGTGGATATGATTTGTCTGGTTGATTCCGATAACGAGCGAGACTTTTATGTTATATTAAATAATATTATTTTAAATTTTATTTAATAATATTTTATTTTAATAACAGATTAATAGTGTTTAACTATTTGAGAGAGAGCAATAACAGGTCTGTGATGCCCTTAGATGTCCGGGGCTGCACGCGCGCTACAAT

>Haplotype_T

ATTGACAGATTGATAGCTCTTTCATGATTTAGTGGTTGGTGGTGCATGGCCGTTCTTAGTTCGTGGATATGATTTGTCTGGTTGATTCCGATAACGAGCGAGACTTTTATGTTATATTAAATAATATTATTATTAAATTTTATTTTTATATAATATTTTATTTTAATAACAGATTAATAGTGTTTAACTATTTGAGAGAGAGCAATAACAGGTCTGTGATGCCCTTAGATGTCCGGGGCTGCACGCGCGCTACAAT

# Appendix D. Fasta sequences of segmented haplotypes

>HVR_4_PART_C_Hap_1_

GATTAATAGTGTTTAACTATTTGAGAGAGAGCAATAACAGGTCTGTGATGCCCTTAGATGTCCGGGGCTGCACGCGCGCTACAAT

>HVR_4_PART_C_Hap_2_

GATTAATAGTGTTTAACTATTTGAGAGAGAGCGATAACGGGTCTGTGATGCCCTTAGATGTCCGGGGCTGCACGCGCGCTACAAT

>HVR_4_PART_C_Hap_3_

GATTAATAGTGTTTAACTATTTGAGAGAGAGCGATAACAGGTCTGTGATGCCCTTAGATGTCCGGGGCTGCACGCGCGCTACAAT

>HVR_4_PART_C_Hap_4_

GATTAATAGTGTTTAACTATTTGAGAGAGAGCGATAACAGGTATGTGATGCCCTTAGATGTCCGGGGCTGCACGCGCGCTACAAT

>HVR_4_PART_B_Hap_1_

CCGATAACGAGCGAGACTTTTATGTTATATTAAATAATATTATTTATAAATTTTATATTTTATATAATATTTTATTTTAATAACA

>HVR_4_PART_B_Hap_2_

CCGATAACGAGCGAGACTTTTATGTTATATTAAATAATATTATTTATAAATTTTATATATTATATAATATTTTATTTTAATAACA

>HVR_4_PART_B_Hap_3_

CCGATAACGAGCGAGACTTTTATGTTATATTAAATAATATTATTATTAAATTTTATTTTTATATAATATTTTATTTTAATAACA

>HVR_4_PART_B_Hap_4_

CCGATAACGAGCGAGACTTTTATGTTATATTAAATAATATTATTATTAAATTTTATTTTATATAATATTTTATTTTAATAACA

>HVR_4_PART_B_Hap_5_

CCGATAACGAGCGAGACTTTTATGTTATATTAAATAATATTATTATTAAATTTTATGGTTTTATATAATATTTTATTTTAATAACA

>HVR_4_PART_B_Hap_6_

CCGATAACGAGCGAGACTTTTATGTTATATTAAATAATATTATTGTTAAATTTTATGGTTTTATATAATATTTTATTTTAATAACA

>HVR_4_PART_B_Hap_7_

CCGATAACGAGCGAGACTTTTATGTTATATTAAATAATATTATTGTTAAATTTTATTTTTATATAATATTTTATTTTAATAACA

>HVR_4_PART_B_Hap_9_

CCGATAACGAGCGAGACTTTTATGTTATATTAAATAATATTATTTATAAATTTTATAGTTTTTATATAATATTTTATTTTAATAACA

>HVR_4_PART_B_Hap_8_

CCGATAACGAGCGAGACTTTTATGTTATATTAAATATAATTATTTTGTTTATTTTAATATAAATAATTAATATTTTAATAACA

>HVR_4_PART_B_Hap_10_

CCGATAACGAGCGAGACTTTTATGTTATATTAAATAATATTATTTTAAATTTTATTTAATAATATTTTATTTTAATAACA

>HVR_4_PART_B_Hap_11_

CCGATAACGAGCGAGACTTTTATGTTATATTAAATATTATTATTTTATTATATAAATAATAATTATTTTAATAACA

>HVR_4_PART_B_Hap_12_

CCGATAACGAGCGAGACTTTTATGTTATATTAAATATTATTATTTTTTATATAAATAATAATTATTTTAATAACA

>HVR_4_PART_B_Hap_13_

CCGATAACGAGCGAGACTTTTATGTTATATTAAATATTATTATTTTATTATTTTATATAAATAATTAATATTTTAATAACA

>HVR_4_PART_B_Hap_14_

CCGATAACGAGCGAGACTTTTATGTTATATTAAATATTATTATTTGTTTATTTTTTATATAAATAATTAATATTTTAATAACA

>HVR_4_PART_B_Hap_15_

CCGATAACGAGCGAGACTTTTATGTTATATTAAATATTATTATTTGTTTATTTTATATAAATAATTAATATTTTAATAACA

>HVR_4_PART_B_Hap_16_

CCGATAACGAGCGAGACTTTTATGTTATATTAAATATTATTATTTGTTTATTTTAATATAAATAATTAATATTTTAATAACA

>HVR_4_PART_B_Hap_17_

CCGATAACGAGCGAGACTTTTATGTTATATTAAATATTATTATTTTGTTTATTTTAATATAAATAATTAATATTTTAATAACA

>HVR_4_PART_A_Hap_1_

ATTGACAGATTGATAGCTCTTTCATGATTTAGTGGTTGGTGGTGCATGGCCGTTCTTAGTTCGTGGATATGATTTGTCTGGTTGATT

>HVR_4_PART_A_Hap_2_

ATTGACAGAGTGATAGCTCTTTCATGATTTAGTGGTTGGTGGTGCATGGCCGTTCTTAGTTCGTGGATATGATTTGTCTGGTTGATT

>HVR_4_PART_A_Hap_3_

ATTGACAGATTGATAGCTCTTTCATGATCTAGTGGTTGGTGGTGCATGGCCGTTCTTAGTTCGTGGATATGATTTGTCTGGTTGATT

>HVR_I_PART_D_Hap_1_

AATATTATTATTAATATTTGGATTGAAAATCTTCAAGTTTAAATAACTTGTTGGTAAAGGAAAGGGCAAGTCTGGTGCCAGCAGCCGCGGTAATACCAGC

>HVR_I_PART_D_Hap_2_

AATATTATTATTAATATTTGGATTGAAAATCTTCAAGTTTAAATAACTTGTTGGTAAAGGTTTGGGCAAGTCTGGTGCCAGCAGCCGCGGTAATACCAGC

>HVR_I_PART_D_Hap_3_

AATATTATTATTAATATTTGGATTGAAAATCTTCAAGTATAAATAACTTGTTGGTAAAGGAAAGGGCAAGTCTGGTGCCAGCAGCCGCGGTAATACCAGC

>HVR_I_PART_D_Hap_4_

AATATTATTATATTAATATTTGGATTGAAAATCTTCAAGTTTAAATAACTTGTTGGTAAAGGAAAGGGCAAGTCTGGTGCCAGCAGCCGCGGTAATACCAGC

>HVR_I_PART_C_Hap_1_

TCGACTCCGGAGAGGGAGCCTGAGAAACGGCTACCACATCCAAGGAAGGCAGCAGGCGCGAAAATTACCCAATTTTAGTTTAAAGAGGTAGTGACGAAAAATGACAACCA

>HVR_I_PART_C_Hap_2_

TCGACTCCGGAGAGGGAGCCTGAGAAACGGCTACCACATCCAAGGAAGGCAGCAGGCGCGAAAATTACCCAATTTTAGTTCAAAGAGGTAGTGACGAAAAATGACAACCA

>HVR_I_PART_C_Hap_3_

TCGACTCCGGAGAGGGAGCCTGAGAAACGGCTACCACATCCAAGGAAGGCAGCAGGCGCGAAAATTACCCAATTTTAGTTAAAAGAGGTAGTGACGAAAAATGACAACCA

>HVR_I_PART_C_Hap_4_

TCGACTCCGGAGAGGGAGCCTGAGAAATGGCTACCACATCCAAGGAAGGCAGCAGGCGCGAAAATTACCCAATTTTAGTTAAAAGAGGTAGTGACGAAAAATGACAACCA

>HVR_I_PART_B_Hap_1_

ATCCTCGCTGATTTTGTTACTAAAACATACCGTATGTGTATCTGGTTTATCAACTTTCGATGGTAGGGTATTGGCCTACCATGGTTGTGACGGATAACGGAGAATTAGGGT

>HVR_I_PART_B_Hap_2_

ATCCTCGCTGATTTTGTTACAAAAACATACCGTATGTGTATCTGGTTTATCAACTTTCGATGGTAGGGTATTGGCCTACCATGGTTGTGACGGATAACGGAGAATTAGGGT

>HVR_I_PART_B_Hap_3_

ATCCTTGCTGATTTTGTTACTAAAACATACCGTATGTGTATCTGGTTTATCAACTTTCGATGGTAGGGTATTGGCCTACCATGGTTGTGACGGATAACGGAGAATTAGGGT

>HVR_I_PART_A_Hap_1_

GTTGGATAACTGAGGTAATTCTTGAGCTAATACACGCTACCTATACCACATTAGTGGTGCGTTTATTTGATTAAACCATTATTTTGGTTGACTCAAAAT

>HVR_I_PART_A_Hap_2_

GTTGGATAACTGAGGTAATTCTTGAGCTAATACACGCTATTTATACCACATTAGTGGTGCGTTTATTTGATTAAACCATTATTTTGGTTGACTCAAAAT

>HVR_I_PART_A_Hap_3_

GTTGGATAACTGACGTAATTCTTGAGCTAATACACGCTATTTATACCACATTAGTGGTGCGTTTATTTGATTAAACCATTATAACGGTTGACTCAAAAT

>HVR_I_PART_A_Hap_4_

GTTGGATAACTGAGGTAATTCTTGAGCTAATACACGCTATTTATACCACATTAGTGGTGCGTTTATTTGATTAAACCATTATAACGGTTGACTCAAAAT

>HVR_I_PART_A_Hap_5_

GTTGGATAACTGAGGTAATTCTTGAGCTAATACACGCTATTTATACCACATTAGTGGTGCGTTTATTTGATTAAACCATTTTAACGGTTGACTCAAAAT

>HVR_I_PART_A_Hap_6_

GTTGGATAACTGAGGTAATTCTTGAGCTAATACACGCTGTTAATACCACATTAGTGGTGCGTTTATTTGATTAAACCATTATATTGGTTGACTCAAAAT

>HVR_I_PART_A_Hap_7_

GTTGGATAACTGAGGTAATTCTTGAGCTAATACACGCTATTTATACCACATTAGTGGTGCGTTTATTTGATTAAACCATTTATACTGGTTGACTCAAAAT

>HVR_I_PART_A_Hap_8_

GTTGGATAACTGAGGTAATTCTTGAGCTAATACACGCTATTTATACCACATTAGTGGTGCGTTTATTTGATTAAACCATTTTATACTGGTTGACTCAAAAT

>HVR_I_PART_A_Hap_9_

GTTGGATAACTGAGGTAATTCTTGAGCTAATACACGCTATTTATACCACATTAGTGGTGCGTTTATTTGATTAAACCATTTTTATATTGGTTGACTCAAAAT

>HVR_I_PART_A_Hap_10_

GTTGGATAACTGAGGTAATTCTTGAGCTAATACACGCTATTTATACCACATTAGTGGTGCGTTTATTTGATTAAACCATTTTATATTGGTTGACTCAAAAT

>HVR_I_PART_A_Hap_11_

GTTGGATAACTGAGGTAATTCTTGAGCTAATACACGCTATTTATACCACATTAGTGGTGCGTTTATTTGATTAAACCATTTTTATATATTGGTTGACTCAAAAT

>HVR_I_PART_A_Hap_12_

GTTGGATAACTGAGGTAATTCTTGAGCTAATACACGCTATTTATACCACATTAGTGGTGCGTTTATTTGATTAAACCATTTATATTGGTTGACTCAAAAT

>HVR_I_PART_A_Hap_13_

GTTGGATAACTGAGGTAATTCTTGAGCTAATACACGCTATTTATACCACATTAGTGGTGCGTTTATTTGATTAAACCATTATATAACGGTTGACTCAAAAT

>COXI_PART_A1_Hap_1_

CCTTTGATGTTAGGT

>COXI_PART_A1_Hap_2_

CCTTTGATGTTGGGT

>COXI_PART_A1_Hap_3_

CCTTTAATATTAGGT

>COXI_PART_A2_Hap_1_

GCTCCTGATATGAGT

>COXI_PART_A2_Hap_2_

GCCCCTGATATGAGT

>COXI_PART_A2_Hap_3_

GCTCCCGATATGAGT

>COXI_PART_A2_Hap_4_

GCACCTGATATGAGT

>COXI_PART_A3_Hap_1_

TTTCCTCGTTTAAAT

>COXI_PART_A3_Hap_2_

TTTCCTCGATTGAAT

>COXI_PART_A3_Hap_3_

TTTCCTCGATTAAAT

>COXI_PART_A3_Hap_4_

TTTCCTCGTTTGAAT

>COXI_PART_A3_Hap_5_

TTTCCTCGATTAATA

>COXI_PART_A3_Hap_6_

TTCCCTCGTCTAAAT

>COXI_PART_A3_Hap_7_

TTCCCTCGTTTGAAT

>COXI_PART_A3_Hap_8_

TTTCCCCGTTTGAAT

>COXI_PART_B1_Hap_1_

AATATTAGTTTTTGA

>COXI_PART_B1_Hap_2_

AACATTAGTTTTTGA

>COXI_PART_B1_Hap_3_

AATATTAGTTTTTGG

>COXI_PART_B1_Hap_4_

AATGTTAGTTTTTGA

>COXI_PART_B2_Hap_1_

TTATTACCGAGTTCT

>COXI_PART_B2_Hap_2_

TTATTGCCGAGTTCT

>COXI_PART_B2_Hap_3_

TTATTACCTAGTTCT

>COXI_PART_B2_Hap_4_

TTATTGCCTAGTTCT

>COXI_PART_B2_Hap_5_

TTGTTGCCTAGTTCT

>COXI_PART_B2_Hap_6_

TTATTGCCAAGTTCT

>COXI_PART_B2_Hap_7_

TTGTTGCCAAGTTCT

>COXI_PART_B2_Hap_8_

CTTTTACCTGCTTCT

>COXI_PART_B2_Hap_9_

TTATTACCTGCTTCT

>COXI_PART_B2_Hap_10_

TTGTTACCTGCCTCT

>COXI_PART_B2_Hap_11_

TTATTACCAAGTTCT

>COXI_PART_B2_Hap_12_

CTATTGCCAAGTTCT

>COXI_PART_B3_Hap_1_

ATGTTTTTACTTTTT

>COXI_PART_B3_Hap_2_

ATTTTTTTGGTTTTT

>COXI_PART_B3_Hap_3_

TTAGTTTTGTTGTTA

>COXI_PART_B3_Hap_4_

ATTTTTTTAGTTTTT

>COXI_PART_B3_Hap_5_

ATTTTCTTAGTTTTT

>COXI_PART_B3_Hap_6_

TTAATGTTATTATTA

>COXI_PART_B3_Hap_7_

TTAATTTTGTTATTA

>COXI_PART_B3_Hap_8_

TTAGTTTTGTTATTA

>COXI_PART_B3_Hap_9_

TTAATTTTGTTGTTA

>COXI_PART_B3_Hap_10_

TTGATTTTGTTATTA

>COXI_PART_B3_Hap_11_

TTGATTTTATTATTA

>COXI_PART_B3_Hap_12_

TTGATTTTGTTATTG

>COXI_PART_B3_Hap_13_

TTGATTTTATTGTTA

>COXI_PART_B3_Hap_14_

TTGGTATTATTATTG

>COXI_PART_B3_Hap_15_

TTGGTTTTATTGTTA

>COXI_PART_B3_Hap_16_

TTGGTTTTGTTATTA

>COXI_PART_B3_Hap_17_

TTGATTTTGTTGTTA

>COXI_PART_B3_Hap_18_

TTGGTATTATTGTTG

>COXI_PART_B3_Hap_19_

TTGGTTTTATTGTTG

>COXI_PART_B3_Hap_20_

TTAGTTTTAGTTGTA

>COXI_PART_B3_Hap_21_

TTAATTTTATTATTA

>COXI_PART_B3_Hap_22_

TTAATGTTGTTATTA

>COXI_PART_C1_Hap_1_

TTGGCTTGTTTTGTT

>COXI_PART_C1_Hap_2_

ACTGCTTGTTTTGTT

>COXI_PART_C1_Hap_3_

ACTGCTTGCTTTGTT

>COXI_PART_C1_Hap_4_

TTAGCTTGTTTTGTT

>COXI_PART_C2_Hap_1_

GATTCGGGTTGTGGA

>COXI_PART_C2_Hap_2_

GATTCAGGTTGTGGA

>COXI_PART_C2_Hap_3_

GATTCAGGTTGTGGT

>COXI_PART_C2_Hap_4_

GATTCTGGTTGTGGT

>COXI_PART_C2_Hap_5_

GATAATGGTCTTGGT

>COXI_PART_C2_Hap_6_

GATAATGGTTTAGGT

>COXI_PART_C2_Hap_7_

GATTCTGGTTGTGGA

>COXI_PART_C3_Hap_1_

ACTAGTTGAACTGTT

>COXI_PART_C3_Hap_2_

ACTAGTTGAACTGTA

>COXI_PART_C3_Hap_3_

ACTAGTTGGACTGTT

>COXI_PART_C3_Hap_4_

ACCAGTTGAACTGTT

>COXI_PART_C3_Hap_5_

ACTAGTTGAACTATT

>COXI_PART_C3_Hap_6_

ACTAGTTGGACTATT

>COXI_PART_C3_Hap_7_

ACTAGTTGAACCGTT

>COXI_PART_D1_Hap_1_

TATCCTCCTTTGTCT

>COXI_PART_D1_Hap_2_

TATCCTCCTCTTTCT

>COXI_PART_D1_Hap_3_

TATCCCCCTTTATCT

>COXI_PART_D1_Hap_4_

TACCCCCCTTTATCT

>COXI_PART_D1_Hap_5_

TACCCTCCTTTATCT

>COXI_PART_D1_Hap_6_

TATCCTCCTTTATCT

>COXI_PART_D1_Hap_7_

TACCCTCCTTTGTCT

>COXI_PART_D1_Hap_8_

TATCCTCCATTGTCT

>COXI_PART_D1_Hap_9_

TATCCTCCGTTGTCT

>COXI_PART_D1_Hap_10_

TATCCACCTTTATCT

>COXI_PART_D1_Hap_11_

TATCTTCCTTTATCT

>COXI_PART_D1_Hap_12_

TATCCCCCTTTGTCT

>COXI_PART_D1_Hap_13_

TACCCCCCCTTGTCT

>COXI_PART_D2_Hap_1_

ACTATGGGTCATCCT

>COXI_PART_D2_Hap_2_

ACTATAGGTCATCCT

>COXI_PART_D2_Hap_3_

ACTTTGGGTCATCCT

>COXI_PART_D2_Hap_4_

ACTATAGGTCATCCA

>COXI_PART_D2_Hap_5_

ACTTTAGGGCATCCT

>COXI_PART_D2_Hap_6_

ACTTTAGGTCATCCT

>COXI_PART_D2_Hap_7_

ACTTTAGGTCATCCC

>COXI_PART_D2_Hap_8_

ACTATGGGTCATCCC

>COXI_PART_D2_Hap_9_

ACCATGGGTCATCCT

>COXI_PART_D2_Hap_10_

ACCATAGGTCATCCT

>COXI_PART_D2_Hap_11_

ACTACGGGTCATCCT

>COXI_PART_D2_Hap_12_

ACTTTAGGACATCCT

>COXI_PART_D2_Hap_13_

ACTTCTGGCCATCCT

>COXI_PART_D2_Hap_14_

ACTTCTGGTCATCCT

>COXI_PART_D2_Hap_15_

ACTTCAGGACATCCT

>COXI_PART_D2_Hap_16_

ACTTCAGGTCACCCT

>COXI_PART_D2_Hap_17_

ACTTCAGGTCATCCT

>COXI_PART_D2_Hap_18_

ACTTCAGGTCATCCA

>COXI_PART_D2_Hap_19_

ACTTCAGGTCATCCG

>COXI_PART_D2_Hap_20_

ACCTCTGGTCATCCT

>COXI_PART_D2_Hap_21_

ACTCTAGGGCATCCT

>COXI_PART_D2_Hap_22_

ACTATAGGGCATCCT

>COXI_PART_D2_Hap_23_

ACTATAGGACATCCT

>COXI_PART_D2_Hap_24_

ACTATGGGGCATCCT

>COXI_PART_D3_Hap_1_

GGGAGAAGTGTTGAT

>COXI_PART_D3_Hap_2_

GGAAGAAGTGTTGAT

>COXI_PART_D3_Hap_3_

GGAAGTAGTGTGGAT

>COXI_PART_D3_Hap_4_

GGAAGTAGTGTTGAT

>COXI_PART_D3_Hap_5_

GGTAGAAGGGTTGAT

>COXI_PART_D3_Hap_6_

GGTTCTAGTGTTGAT

>COXI_PART_D3_Hap_7_

GGCTCTAGTGTTGAT

>COXI_PART_D3_Hap_8_

GGTTCTAGTGTAGAT

>COXI_PART_D3_Hap_9_

GGTAGGAGTGTTGAT

>COXI_PART_D3_Hap_10_

GGTAGAAGTGTTGAT

>COXI_PART_D3_Hap_11_

GGGAGTAGTGTTGAT

>COXI_PART_D3_Hap_12_

GGAAGAAGGGTTGAT

>COXI_PART_E1_Hap_1_

TTGGCAATTTTTGCT

>COXI_PART_E1_Hap_2_

TTGGCTATCTTTGCT

>COXI_PART_E1_Hap_3_

TTAGCTATCTTTGCT

>COXI_PART_E1_Hap_4_

TTGGCTATCTTTGCC

>COXI_PART_E1_Hap_5_

TTGTCTATCTTTGCC

>COXI_PART_E1_Hap_6_

CTTGCTGTTTTTAGT

>COXI_PART_E1_Hap_7_

TTAGCTATTTTTGCT

>COXI_PART_E1_Hap_8_

TTGGCTATTTTTGCT

>COXI_PART_E1_Hap_9_

CTTGCTATTTTTGCT

>COXI_PART_E1_Hap_10_

CTGGCTATTTTTGCT

>COXI_PART_E1_Hap_11_

TTGGCTATTTTCAGT

>COXI_PART_E1_Hap_12_

TTGGCTATTTTTAGT

>COXI_PART_E1_Hap_13_

TTAGCAATTTTTGCT

>COXI_PART_E1_Hap_14_

CTTGCTATTTTTAGT

>COXI_PART_E2_Hap_1_

TTACATTTGGCTGGT

>COXI_PART_E2_Hap_2_

TTACATTTAGCTGGT

>COXI_PART_E2_Hap_3_

TTGCATTTAGCTGGT

>COXI_PART_E2_Hap_4_

TTGCATTTGGCTGGT

>COXI_PART_E2_Hap_5_

TTCCATTTGGCTGGT

>COXI_PART_E2_Hap_6_

TTACATCTTTCTGGT

>COXI_PART_E2_Hap_7_

TTGCACCTTTCTGGT

>COXI_PART_E2_Hap_8_

TTACATTTAGCCGGT

>COXI_PART_E2_Hap_9_

TTGCATCTTTCTGGT

>COXI_PART_E3_Hap_1_

GTTAGTTCTATTTTG

>COXI_PART_E3_Hap_2_

ATTAGTTCTATTTTA

>COXI_PART_E3_Hap_3_

ATTAGTTCTATTTTG

>COXI_PART_E3_Hap_4_

GTAAGTTCTATTTTA

>COXI_PART_E3_Hap_5_

GTGAGTTCCATTTTA

>COXI_PART_E3_Hap_6_

GTTAGTTCCATTTTA

>COXI_PART_E3_Hap_7_

GTTAGTTCTATCTTG

>COXI_PART_E3_Hap_8_

GTAAGTTCTATTTTG

>COXI_PART_E3_Hap_9_

GTAAGTTCTATTCTA

>COXI_PART_E3_Hap_10_

GTGAGTTCTATCTTA

>COXI_PART_E3_Hap_11_

GTTAGTTCTATTTTA

>COXI_PART_E3_Hap_12_

ATTAGTTCAATTTTA

>COXI_PART_F1_Hap_1_

GGTGCTATTAATTTT

>COXI_PART_F1_Hap_2_

GGTGCTATTAACTTT

>COXI_PART_F1_Hap_3_

GGTGGTATTAATTTT

>COXI_PART_F1_Hap_4_

GGTGCTATCAATTTT

>COXI_PART_F1_Hap_5_

GGTGCCATTAATTTT

>COXI_PART_F1_Hap_6_

GGTGGTATCAATTTT

>COXI_PART_F2_Hap_1_

ATGTGTACTACAAAA

>COXI_PART_F2_Hap_2_

ATGTGTACTGTTAAG

>COXI_PART_F2_Hap_3_

ATGTGTACTACTAAA

>COXI_PART_F2_Hap_4_

ATGTGTACTGTTAAA

>COXI_PART_F2_Hap_5_

ATGTGTACTATTAAA

>COXI_PART_F2_Hap_6_

ATGTGTACGACAAAG

>COXI_PART_F2_Hap_7_

ATGTGTACTACAAAG

>COXI_PART_F2_Hap_8_

ATGTGTACCACTAAA

>COXI_PART_F2_Hap_9_

ATGTGTACTACGAAA

>COXI_PART_F2_Hap_10_

ATGTGTACTATTAAG

>COXI_PART_F2_Hap_11_

ATATGTACTACTAAG

>COXI_PART_F2_Hap_12_

ATATGTACTACTAAA

>COXI_PART_F2_Hap_13_

ATGTGTACTCCAAAA

>COXI_PART_F3_Hap_1_

AATCTTCGTAGTTCT

>COXI_PART_F3_Hap_2_

AATTTACGTTCTGGT

>COXI_PART_F3_Hap_3_

AATTTACGTTCTAGT

>COXI_PART_F3_Hap_4_

AATTTGCGTTCTAGT

>COXI_PART_F3_Hap_5_

AATTTGCGCTCTAGT

>COXI_PART_F3_Hap_6_

AATCTTCGCAGTTCT

>COXI_PART_G1_Hap_1_

TCTATTAGTCTAGAG

>COXI_PART_G1_Hap_2_

TCTATTAGTTTAGAG

>COXI_PART_G1_Hap_3_

TCAATTAGTTTAGAG

>COXI_PART_G1_Hap_4_

TCTATTAGTTTGGAG

>COXI_PART_G1_Hap_5_

TCAATTAGTCTAGAG

>COXI_PART_G1_Hap_6_

TCAATTAGTCTGGAG

>COXI_PART_G1_Hap_7_

TCAATTAGTTTAGAA

>COXI_PART_G1_Hap_8_

TCTGTTTCTCTTAAT

>COXI_PART_G1_Hap_9_

TCTGTTTCTCTCAAT

>COXI_PART_G1_Hap_10_

TCTGTCTCTCTTAAT

>COXI_PART_G1_Hap_11_

TCAATTAGTTTGGAG

>COXI_PART_G1_Hap_12_

TCGATTAGTTTAGAG

>COXI_PART_G1_Hap_13_

TCAATTAGCTTAGAG

>COXI_PART_G1_Hap_14_

TCTATTAGTTTAGAA

>COXI_PART_G2_Hap_1_

CATATGAGTTTGTTT

>COXI_PART_G2_Hap_2_

CATATGAGTTTATTT

>COXI_PART_G2_Hap_3_

CATATAAGTTTGTTT

>COXI_PART_G2_Hap_4_

CATATAAGATTATTT

>COXI_PART_G2_Hap_5_

CATATGAGATTATTT

>COXI_PART_G2_Hap_6_

AATATGAGTTTATTT

>COXI_PART_G2_Hap_7_

AATATGAGTTTGTTT

>COXI_PART_G2_Hap_8_

AATATAAGTTTGTTT

>COXI_PART_G2_Hap_9_

CATATAAGTTTATTT

>COXI_PART_G2_Hap_10_

AATATAAGTTTATTT

>COXI_PART_G2_Hap_11_

AACATAAGTTTGTTT

>COXI_PART_G3_Hap_1_

GTTTGGACTGTGTTT

>COXI_PART_G3_Hap_2_

GTTTGGACTGTATTT

>COXI_PART_G3_Hap_3_

GTTTGAACTGTGTTT

>COXI_PART_G3_Hap_4_

GTTTGGACTGTTTTT

>COXI_PART_G3_Hap_5_

ATTTGAACTATTTTT

>COXI_PART_G3_Hap_6_

ATTTGGACTATTTTT

>COXI_PART_G3_Hap_7_

GTTTGAACAGTGTTT

>COXI_PART_G3_Hap_8_

GTTTGAACGGTGTTT

>COXI_PART_G3_Hap_9_

GTTTGGACGGTGTTT

>COXI_PART_G3_Hap_10_

GTTTGGGCTGTGTTT

>COXI_PART_G3_Hap_11_

GTTTGAACGGTATTT

>COXI_PART_G3_Hap_12_

GTTTGGACAGTGTTT

>COXI_PART_H1_Hap_1_

GTTACTGTGTTTTTG

>COXI_PART_H1_Hap_2_

GTTACGGTGTTTTTA

>COXI_PART_H1_Hap_3_

GTTACAGTGTTTTTA

>COXI_PART_H1_Hap_4_

GTTACGGTGTTTTTG

>COXI_PART_H1_Hap_5_

GTTACTGTTTTTTTA

>COXI_PART_H1_Hap_6_

GTTACAGTGTTTTTG

>COXI_PART_H1_Hap_7_

GTTACTGTATTTTTG

>COXI_PART_H1_Hap_8_

GTTACAGTTTTTTTA

>COXI_PART_H1_Hap_9_

GTAACGGTGTTTTTA

>COXI_PART_H1_Hap_10_

GTAACGGTGTTCTTA

>COXI_PART_H1_Hap_11_

GTAACAGTGTTTTTA

>COXI_PART_H1_Hap_12_

GTTACTGTCTTTTTA

>COXI_PART_H1_Hap_13_

GTAACAGTGTTCTTA

>COXI_PART_H1_Hap_14_

GTTACAGTATTTTTG

>COXI_PART_H1_Hap_15_

GTTACAGTATTTTTA

>COXI_PART_H2_Hap_1_

TTGGTTTTGTCTTTA

>COXI_PART_H2_Hap_2_

TTGGTTTTATCTTTA

>COXI_PART_H2_Hap_3_

TTAGTACTTTCTTTA

>COXI_PART_H2_Hap_4_

TTAGTTCTTTCTTTA

>COXI_PART_H2_Hap_5_

TTGGTTCTTTCTTTA

>COXI_PART_H2_Hap_6_

TTAGTGCTTTCTTTA

>COXI_PART_H2_Hap_7_

TTGGTTCTCTCTTTA

>COXI_PART_H2_Hap_8_

TTAGTTCTTTCTTTG

>COXI_PART_H2_Hap_9_

TTGGTACTTTCTTTG

>COXI_PART_H2_Hap_10_

TTGGTACTTTCTTTA

>COXI_PART_H2_Hap_11_

CTGGTACTTTCTTTG

>COXI_PART_H2_Hap_12_

TTAGTGCTTTCTTTG

>COXI_PART_H2_Hap_13_

TTGGTGCTTTCTTTG

>COXI_PART_H2_Hap_14_

TTGGTGCTTTCTTTA

>COXI_PART_H2_Hap_15_

TTAGTACTTTCTTTG

>COXI_PART_H2_Hap_16_

TTGGTGCTTTCCTTA

>COXI_PART_H2_Hap_17_

TTAGTTCTTTCTCTG

>COXI_PART_H2_Hap_18_

TTGGTTTTGTCTTTG

>COXI_PART_H2_Hap_19_

TTGGTCTTGTCTTTA

>COXI_PART_H3_Hap_1_

CCTGTTTTAGCTGGT

>COXI_PART_H3_Hap_2_

CCTGTTTTAGCAGGT

>COXI_PART_H3_Hap_3_

CCTGTGTTGGCTGGT

>COXI_PART_H3_Hap_4_

CCTGTATTGGCTGGT

>COXI_PART_H3_Hap_5_

CCTGTATTAGCTGGT

>COXI_PART_H3_Hap_6_

CCTGTTTTAGCTAGT

>COXI_PART_H3_Hap_7_

CCTGTTTTAGCGGGT

>COXI_PART_H3_Hap_8_

CCTGTTTTGGCAGGT

>COXI_PART_H3_Hap_9_

CCTGTATTAGCAGGT

>COXI_PART_H3_Hap_10_

CCTGTATTAGCGGGT

>COXI_PART_I1_Hap_1_

GCTATTACTATGTTG

>COXI_PART_I1_Hap_2_

GCTATTACTATGTTA

>COXI_PART_I1_Hap_3_

GCTATTACTATATTA

>COXI_PART_I1_Hap_4_

GCTATTACTATATTG

>COXI_PART_I1_Hap_5_

GCCATTACTATGTTA

>COXI_PART_I1_Hap_6_

GCTATTACTATGCTT

>COXI_PART_I1_Hap_7_

GCTATTACTATACTT

>COXI_PART_I2_Hap_1_

TTATTTGATCGCAAT

>COXI_PART_I2_Hap_2_

TTATTTGATCGTAAT

>COXI_PART_I2_Hap_3_

TTGTTTGATCGTAAT

>COXI_PART_I2_Hap_4_

TTGGTTGATCGAAAT

>COXI_PART_I2_Hap_5_

TTATTCGATCGTAAT

>COXI_PART_I2_Hap_6_

TTAATTGATCGTAAT

>COXI_PART_I2_Hap_7_

TTGATTGATCGTAAT

>COXI_PART_I3_Hap_1_

TTTAATACTTCTTTC

>COXI_PART_I3_Hap_2_

TTTAATACTTCTTTT

>COXI_PART_I3_Hap_3_

TTTAATGGTTCTTTT

>COXI_PART_J1_Hap_1_

TTTGATCCTAGTGCT

>COXI_PART_J1_Hap_2_

TTTGACCCAAGTGCT

>COXI_PART_J1_Hap_3_

TTTGATCCAAGTGCT

>COXI_PART_J1_Hap_4_

TTTGATCCTAGTTCT

>COXI_PART_J1_Hap_5_

TTTGACCCAAGTGCC

>COXI_PART_J1_Hap_6_

TTTGATCCGAGTGCC

>COXI_PART_J1_Hap_7_

TTTGATCCTAGGGCT

>COXI_PART_J1_Hap_8_

TTTGATCCTAGAGCT

>COXI_PART_J1_Hap_9_

TTTGATCCTAGTTTT

>COXI_PART_J1_Hap_10_

TTTGATCCTAGTTTC

>COXI_PART_J2_Hap_1_

GGTGGTAATCCTTTG

>COXI_PART_J2_Hap_2_

GGTGGTAATCCTCTT

>COXI_PART_J2_Hap_3_

GGTGGTAATCCTTTA

>COXI_PART_J2_Hap_4_

GGAGGTAATCCTTTG

>COXI_PART_J2_Hap_5_

GGGGGTAATCCTTTG

>COXI_PART_J2_Hap_6_

GGTGGTAATCCTCTG

>COXI_PART_J3_Hap_1_

ATTTATCAACATTTA

>COXI_PART_J3_Hap_2_

ATTTATCAACATTTG

>COXI_PART_J3_Hap_3_

ATTTATCAGCATTTA

>COXI_PART_J3_Hap_4_

ATTTATCAGCATTTG

>COXI_PART_J3_Hap_5_

GTTTATCAACATTTG

>COXI_PART_J3_Hap_6_

ATTTATCAACATCTT

>COXI_PART_K1_Hap_1_

TTTTGGTTCTTTGGT

>COXI_PART_K1_Hap_2_

TTCTGATTCTTTGGT

>COXI_PART_K1_Hap_3_

TTTTGATTCTTTGGA

>COXI_PART_K1_Hap_4_

TTTTGATTCTTTGGC

>COXI_PART_K1_Hap_5_

TTTTGGTTTTTTGGA

>COXI_PART_K1_Hap_6_

TTTTGGTTTTTTGGG

>COXI_PART_K1_Hap_7_

TTTTGGTTTTTTGGT

>COXI_PART_K1_Hap_8_

TTTTGGTTTTTTGGC

>COXI_PART_K1_Hap_9_

TTTTGATTTTTTGGT

>COXI_PART_K1_Hap_10_

TTTTGGTTTTTCGGT

>COXI_PART_K1_Hap_11_

TTTTGATTCTTTGGT

>COXI_PART_K2_Hap_1_

CATCCTGAGGTATAT

>COXI_PART_K2_Hap_2_

CATCCGGAAGTTTAT

>COXI_PART_K2_Hap_3_

CATCCAGAAGTTTAT

>COXI_PART_K2_Hap_4_

CATCCTGAGGTTTAT

>COXI_PART_K2_Hap_5_

CATCCTGAAGTTTAT

>COXI_PART_K2_Hap_6_

CATCCCGAAGTATAT

>COXI_PART_K2_Hap_7_

CATCCCGAGGTTTAT

>COXI_PART_K2_Hap_8_

CATCCTGAAGTATAT

>COXI_PART_K3_Hap_1_

ATTTTAATTCTTCCT

>COXI_PART_K3_Hap_2_

ATTTTGATTTTACCT

>COXI_PART_K3_Hap_3_

ATTTTGATTCTTCCT

>COXI_PART_K3_Hap_4_

ATCTTAATTCTTCCT

>COXI_PART_K3_Hap_5_

ATTTTAATTTTGCCT

>COXI_PART_K3_Hap_6_

ATTTTAATTTTACCT

>COXI_PART_L1_Hap_1_

GCTTTTGGTATTATT

>COXI_PART_L1_Hap_2_

GCTTTTGGAATTATT

>COXI_PART_L1_Hap_3_

GCTTTCGGTATTATT

>COXI_PART_L1_Hap_4_

GCTTTTGGAATTGTT

>COXI_PART_L1_Hap_5_

GCTTTTGGTATCATT

>COXI_PART_L2_Hap_1_

AGTCAAAGTACTCTT

>COXI_PART_L2_Hap_2_

AGTCAGTGTACTCTT

>COXI_PART_L2_Hap_3_

AGGCAAAGTACTCTT

>COXI_PART_L2_Hap_4_

AGTCAAAGTACTTTA

>COXI_PART_L2_Hap_5_

AGTCAAAGTACTCTC

>COXI_PART_L2_Hap_6_

AGTCAATGTACTTTA

>COXI_PART_L2_Hap_7_

AGTCAATGTACTTTG

>COXI_PART_L2_Hap_8_

AGTCAGTGTACTTTA

>COXI_PART_L2_Hap_9_

AGACAAAGTACTCTT

>COXI_PART_L2_Hap_10_

AGTCAATGTACCTTG

>COXI_PART_L3_Hap_1_

TATTTAACTGGTAAA

>COXI_PART_L3_Hap_2_

TATTTGACTGGTAAA

>COXI_PART_L3_Hap_3_

TATCTAACTGGTAAG

>COXI_PART_L3_Hap_4_

TATTTAACTGGTAAG

>COXI_PART_L3_Hap_5_

TATTTGACTGGTAAG

>COXI_PART_L3_Hap_6_

TATTTAACCGGTAAA

>COXI_PART_L3_Hap_7_

TACTTGACTGGTAAA

>COXI_PART_L3_Hap_8_

TATCTAACTGGTAAA

>COXI_PART_L3_Hap_9_

TACTTAACTGGTAAA

>COXI_PART_M1_Hap_1_

AAGGAGGTTTTTGGT

>COXI_PART_M1_Hap_2_

AAAGAAGTTTTTGGT

>COXI_PART_M1_Hap_3_

AAGGAAGTCTTTGGT

>COXI_PART_M1_Hap_4_

AAGGAGGTATTTGGT

>COXI_PART_M1_Hap_5_

AAGGAGGTGTTTGGT

>COXI_PART_M1_Hap_6_

AAAGAGGTGTTTGGT

>COXI_PART_M1_Hap_7_

AAGGAAGTATTTGGT

>COXI_PART_M1_Hap_8_

AAGGAAGTTTTTGGT

>COXI_PART_M1_Hap_9_

AAAGAGGTTTTTGGT

>COXI_PART_M1_Hap_10_

AAGGAAGTGTTTGGT

>COXI_PART_M1_Hap_11_

AAAGAGGTATTTGGT

>COXI_PART_M1_Hap_12_

AAGGAGGTCTTTGGT

>COXI_PART_M2_Hap_1_

ACTTTGGGTATGATT

>COXI_PART_M2_Hap_2_

ACTTTAGGTATGATT

>COXI_PART_M2_Hap_3_

ACCTTAGGTATAATT

>COXI_PART_M2_Hap_4_

ACCTTGGGTATAATT

>COXI_PART_M2_Hap_5_

ACTCTAGGTATGATT

>COXI_PART_M2_Hap_6_

TATCTTGGTATGGTT

>COXI_PART_M2_Hap_7_

ACTTTAGGTATAATT

>COXI_PART_M2_Hap_8_

ACTTTGGGTATAATT

>COXI_PART_M2_Hap_9_

TATTTAGGTATGGTT

>COXI_PART_M2_Hap_10_

TATTTGGGTATGGTT

>COXI_PART_M2_Hap_11_

TATTTAGGAATGGTT

>COXI_PART_M2_Hap_12_

ACTCTGGGTATGATT

>COXI_PART_M2_Hap_13_

ACTCTTGGTATGATT

>COXI_PART_M3_Hap_1_

TATGCTATTTTGAGA

>COXI_PART_M3_Hap_2_

TATGCTATTTTAAGA

>COXI_PART_M3_Hap_3_

TATGCTATTTTAAGT

>COXI_PART_M3_Hap_4_

TATGCAATTTTAAGT

>COXI_PART_M3_Hap_5_

TATGCGATTTTAAGT

>COXI_PART_M3_Hap_6_

TATGCAATTTTGAGT

>COXI_PART_M3_Hap_7_

TACGCGATTTTAAGT

>COXI_PART_M3_Hap_8_

TATGCTATTCTAAGT

>COXI_PART_M3_Hap_9_

TATGCTATTTTGAGT

>COXI_PART_M3_Hap_10_

TACGCTATCTTAAGT

>COXI_PART_M3_Hap_11_

TACGCTATTTTAAGT

>COXI_PART_M3_Hap_12_

TATGCTATTTTAAGG

>COXI_PART_M3_Hap_13_

TACGCTATTTTAAGA

>COXI_PART_N1_Hap_1_

ATTGGTTTAATTGGT

>COXI_PART_N1_Hap_2_

ATTGGTTTGATTGGT

>COXI_PART_N1_Hap_3_

ATTGGTTTAATCGGT

>COXI_PART_N1_Hap_4_

ATTGGATTGATTGGT

>COXI_PART_N1_Hap_5_

ATTGGATTAATTGGT

>COXI_PART_N1_Hap_6_

ATTGGGTTAATTGGT

>COXI_PART_N2_Hap_1_

TGTGTTGTTTGGGCT

>COXI_PART_N2_Hap_2_

TGTGTAGTATGGGCT

>COXI_PART_N2_Hap_3_

TGTGTAGTGTGGGCT

>COXI_PART_N2_Hap_4_

TGTGTAGTTTGAGCT

>COXI_PART_N2_Hap_5_

TGTGTAGTTTGGGCT

>COXI_PART_N2_Hap_6_

TGTGTTGTCTGGGCT

>COXI_PART_N2_Hap_7_

TGTGTTGTTTGAGCT

>COXI_PART_N2_Hap_8_

TGTGTTGTTTGGGCC

>COXI_PART_N2_Hap_9_

TGTGTGGTTTGGGCT

>COXI_PART_N3_Hap_1_

CATCACATGTATACT

>COXI_PART_N3_Hap_2_

CATCACATATATACT

>COXI_PART_N3_Hap_3_

CATCATATGTATACT

>COXI_PART_N3_Hap_4_

CATCATATATATACT

>COXI_PART_N3_Hap_5_

CATCATATGTATACC

>COXI_PART_N3_Hap_6_

CACCATATGTATACT

>COXI_PART_O1_Hap_1_

GTTGGTATGGATATT

>COXI_PART_O1_Hap_2_

GTTGGTATAGATTTT

>COXI_PART_O1_Hap_3_

GTTGGTATGGATTTT

>COXI_PART_O1_Hap_4_

GTTGGTATGGATTTC

>COXI_PART_O1_Hap_5_

GTTGGTATGGATATC

>COXI_PART_O1_Hap_6_

GTTGGGATGGATATT

>COXI_PART_O1_Hap_7_

GTTGGTATAGATATT

>COXI_PART_O1_Hap_8_

GTTGGTATGGACATT

>COXI_PART_O1_Hap_9_

GTTGGAATGGATTTT

>COXI_PART_O1_Hap_10_

GTTGGAATGGATTTC

>COXI_PART_O1_Hap_11_

GTGGGTATAGATATT

>COXI_PART_O1_Hap_12_

GTTGGTATATATTTT

>COXI_PART_O2_Hap_1_

GATTCTCGTGCTTAT

>COXI_PART_O2_Hap_2_

GACTCTCGTGCTTAT

>COXI_PART_O2_Hap_3_

GATTCCCGTGCTTAT

>COXI_PART_O3_Hap_1_

TTTACTGCTGCCACT

>COXI_PART_O3_Hap_2_

TTTACTGCTGCTACT

>COXI_PART_O3_Hap_3_

TTTACTGCTGCTACG

>COXI_PART_O3_Hap_4_

TTTACTGCTGCCACG

>COXI_PART_O3_Hap_5_

TTTACTGCTGCTACC

>COXI_PART_O3_Hap_6_

TTTACTGCTGCAACT

>COXI_PART_O3_Hap_7_

TTTCCTGCTGCAACT

>COXI_PART_O3_Hap_8_

TTTACTGCTGCGACT

>COXI_PART_O3_Hap_9_

TTTACCGCTGCTACT

>COXI_PART_P1_Hap_1_

ATAGTTATTGCTGTT

>COXI_PART_P1_Hap_2_

ATAGTGATTGCTGTC

>COXI_PART_P1_Hap_3_

ATAGTGATTGCTGTT

>COXI_PART_P1_Hap_4_

ATAATTATTGCTGTG

>COXI_PART_P1_Hap_5_

ATGATTATTGCTGTA

>COXI_PART_P1_Hap_6_

ATAATTATTGCTGTA

>COXI_PART_P1_Hap_7_

ATGATTATTGCAGTT

>COXI_PART_P1_Hap_8_

ATAGTAATTGCTGTT

>COXI_PART_P1_Hap_9_

ATAATTATTGCAGTT

>COXI_PART_P1_Hap_10_

ATGATTATTGCTGTG

>COXI_PART_P1_Hap_11_

ATGATTATTGCTGTT

>COXI_PART_P1_Hap_12_

ATCATTATTGCTGTA

>COXI_PART_P2_Hap_1_

CCTACTGGTGTAAAG

>COXI_PART_P2_Hap_2_

CCTACAGGTGTTAAA

>COXI_PART_P2_Hap_3_

CCTACAGGTGTTAAG

>COXI_PART_P2_Hap_4_

CCTACCGGTGTAAAG

>COXI_PART_P2_Hap_5_

CCTACAGGTGTAAAG

>COXI_PART_P2_Hap_6_

CCTACGGGTGTAAAG

>COXI_PART_P2_Hap_7_

CCTACTGGAGTTAAG

>COXI_PART_P2_Hap_8_

CCTACTGGGGTTAAG

>COXI_PART_P2_Hap_9_

CCTACTGGTGTTAAG

>COXI_PART_P2_Hap_10_

CCTACTGGTGTAAAA

>COXI_PART_P2_Hap_11_

CCCACTGGTGTAAAG

>COXI_PART_P2_Hap_12_

CCAACTGGTGTAAAG

>COXI_PART_P2_Hap_13_

CCTACAGGTGTAAAA

>COXI_PART_P2_Hap_14_

CCTACGGGTGTTAAA

>COXI_PART_P3_Hap_1_

GTGTTTAGTTGATTG

>COXI_PART_P3_Hap_2_

GTGTTTAGTTGGTTG

>COXI_PART_P3_Hap_3_

GTGTTTAGTTGATTA

>COXI_PART_P3_Hap_4_

GTTTTTAGTTGGTTA

>COXI_PART_P3_Hap_5_

GTATTTAGTTGATTG

>COXI_PART_P3_Hap_6_

GTATTTAGTTGATTA

>COXI_PART_P3_Hap_7_

GTTTTTAGATGGTTA

>COXI_PART_P3_Hap_8_

GTTTTTAGTTGATTG

>COXI_PART_Q1_Hap_1_

GCTACTTTGTTTGGT

>COXI_PART_Q1_Hap_2_

GCTACTTTATTTGGT

>COXI_PART_Q1_Hap_3_

GCTACTTTTTTTGGT

>COXI_PART_Q1_Hap_4_

GCTACTTTGTTTGGA

>COXI_PART_Q2_Hap_1_

TCACGTTTGGTTTTT

>COXI_PART_Q2_Hap_2_

TCACGTTTAGTTTTT

>COXI_PART_Q2_Hap_3_

TCGCGTTTGGTTTTT

>COXI_PART_Q2_Hap_4_

ACTGTTATAGTTTAT

>COXI_PART_Q2_Hap_5_

ACTGTTATAGTTTAC

>COXI_PART_Q3_Hap_1_

CAACCAGTATTATTG

>COXI_PART_Q3_Hap_2_

CAACCAGTATTATTA

>COXI_PART_Q3_Hap_3_

CAACCAGTATTGTTA

>COXI_PART_Q3_Hap_4_

CAACCAGTGTTATTG

>COXI_PART_Q3_Hap_5_

CAACCAGTGTTATTA

>COXI_PART_Q3_Hap_6_

CAACCTTTATTATTT

>COXI_PART_Q3_Hap_7_

CAACCTTTGTTATTT

>COXI_PART_R1_Hap_1_

TGAGTAATTGGTTTT

>COXI_PART_R1_Hap_2_

TGAGTTATAGGTTTT

>COXI_PART_R1_Hap_3_

TGGGTAATTGGTTTT

>COXI_PART_R1_Hap_4_

TGGGTGATTGGTTTT
