# Supplementary material for: Genetic characterization of Strongyloides fuelleborni infecting free-roaming African vervets (Chlorocebus aethiops sabaeus) on the Caribbean island of St. Kitts
Source: Int J Parasitol Parasites Wildl. 2023 Feb 16;20:153–61. doi: 10.1016/j.ijppaw.2023.02.003 (PMC9969202; doi:10.1016/j.ijppaw.2023.02.003)

Tree A - This is the same tree shown in Figure 2 of the main manuscript, though with tip labels (isolate names) provided.

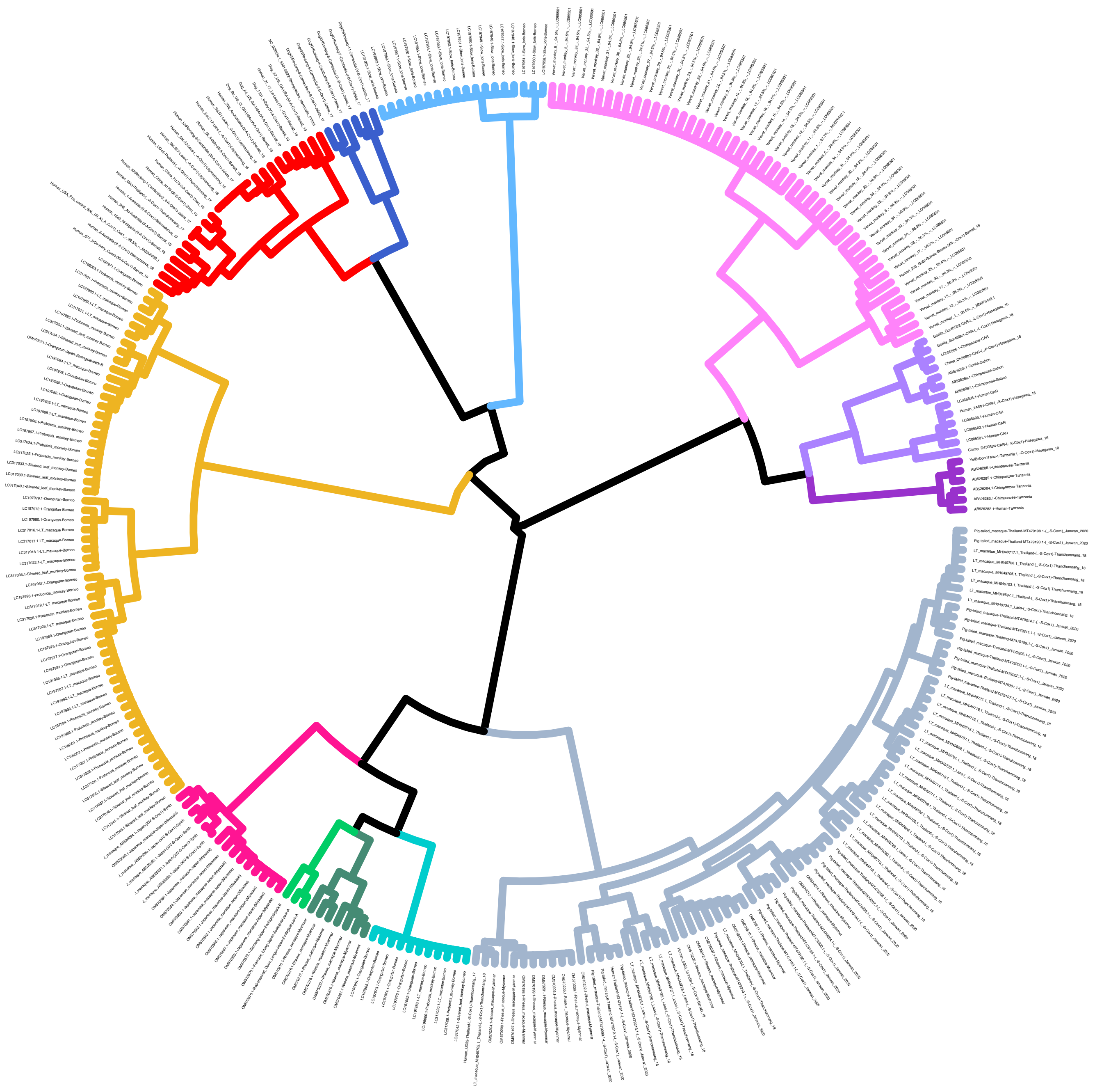

Tree B - This is the same tree shown in Figure 3 of the main manuscript, though with tip labels (isolate names) provided.

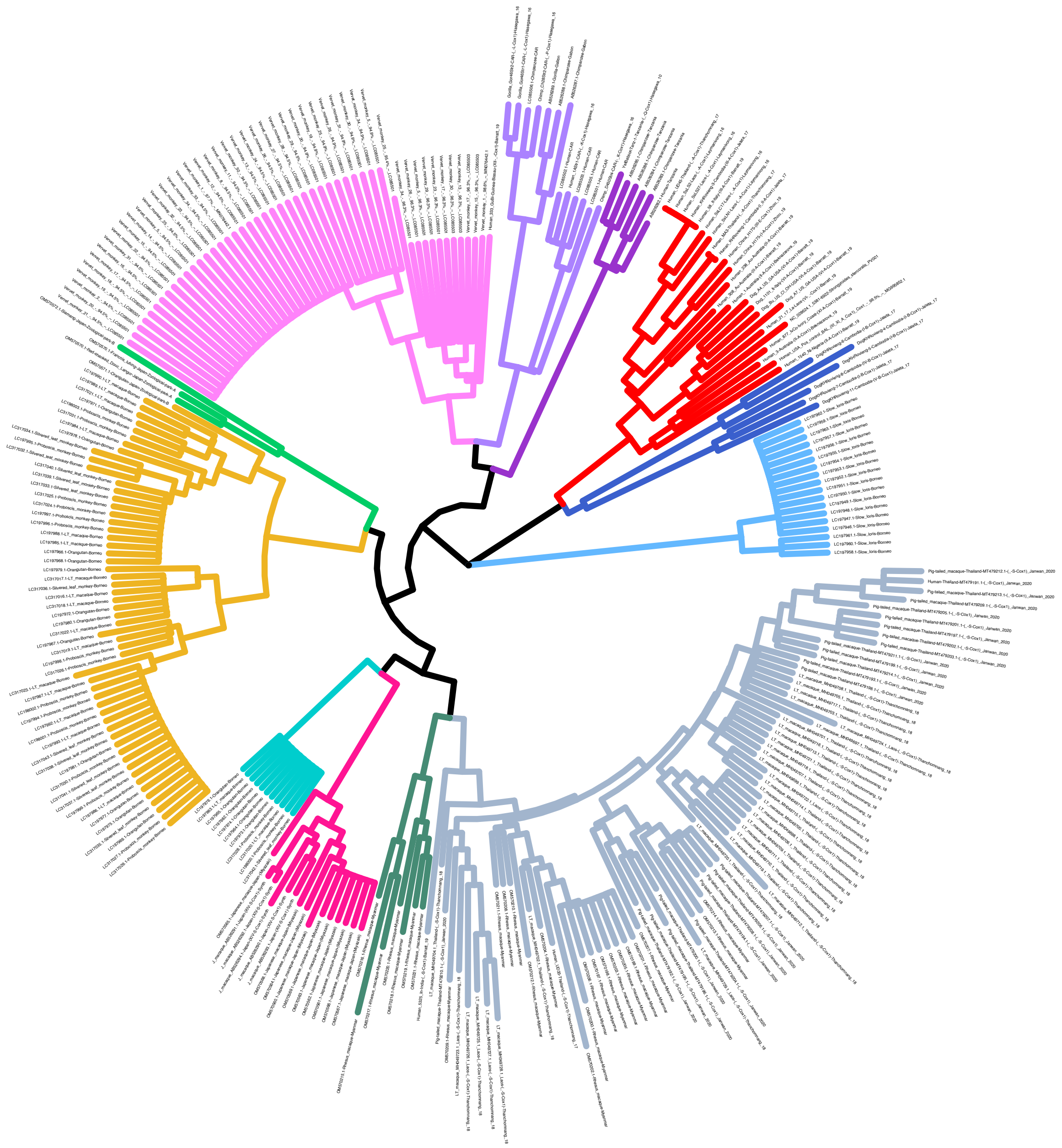

Supplement: Multimedia component 3 [file mmc3.pdf]
